# Echinacoside reduces intracellular c-di-GMP levels and potentiates tobramycin activity against *Pseudomonas aeruginosa* biofilm aggregates

**DOI:** 10.1038/s41522-025-00673-2

**Published:** 2025-03-07

**Authors:** Yu-Ming Cai, Feng Hong, Amber De Craemer, Jacob George Malone, Aurélie Crabbé, Tom Coenye

**Affiliations:** 1 Laboratory of Pharmaceutical Microbiology, Ghent University, Ghent, Belgium; 2 Group of Microbiological Engineering and Biomedical Materials, College of Biological Science and Medical Engineering, Donghua University, North Ren Min Road 2999, 201620 Shanghai, China; 3 National Advanced Functional Fiber Innovation Centre, Wu Jiang, Su Zhou, China; 4 John Innes Centre, Norwich Research Park, Colney Lane, Norwich, UK; 5 School of Biological Sciences, University of East Anglia, Norwich, UK; 6 Present Address: John Innes Centre, Norwich Research Park, Colney Lane, Norwich, UK

**Keywords:** Microbiology, Biofilms

## Abstract

Cyclic diguanylate (c-di-GMP) is a central biofilm regulator in *Pseudomonas aeruginosa*, where increased intracellular levels promote biofilm formation and antibiotic tolerance. Targeting the c-di-GMP network may be a promising anti-biofilm approach, but most strategies studied so far aimed at eliminating surface-attached biofilms, while in vivo *P. aeruginosa* biofilms often occur as suspended aggregates. Here, the expression profile of c-di-GMP metabolism-related genes was analysed among 32 *P. aeruginosa* strains grown as aggregates in synthetic cystic fibrosis sputum. The diguanylate cyclase SiaD proved essential for auto-aggregation under in vivo-like conditions. Virtual screening predicted a high binding affinity of echinacoside towards the active site of SiaD. Echinacoside reduced c-di-GMP levels and aggregate sizes and potentiated tobramycin activity against aggregates in >80% of strains tested. This synergism was also observed in *P. aeruginosa*-infected 3-D alveolar epithelial cells and murine lungs, demonstrating echinacoside’s potential as an adjunctive therapy for recalcitrant *P. aeruginosa* infections.

## Introduction

It is estimated that biofilms, surface-attached or suspended multicellular bacterial communities embedded in a self-produced matrix, are associated with more than 80% of chronic infections^1,2^. The secretion of extracellular polymers (including polysaccharides, proteins, and DNA), reduced metabolic activity, and an altered microenvironment all contribute to the reduced susceptibility of biofilm-associated microorganisms towards antimicrobial agents and host responses^3–5^ and render the eradication of chronic infections difficult at best^6^. With very few new antibiotics coming to the market^7,8^ and the lack of clinically relevant anti-biofilm activity of many antibiotics, novel strategies that enhance the susceptibility of biofilms toward antibiotics are urgently needed^9^.

Different therapeutic approaches are under development to either interfere with crucial structural traits of biofilms or modulate signalling pathways that regulate biofilm formation and dispersal^10,11^. The intracellular secondary messenger cyclic di-guanosine monophosphate (c-di-GMP) emerged as a promising target due to its central role in biofilm regulation and wide distribution among bacterial species^12,13^. An increased production of c-di-GMP promotes biofilm formation and maturation through multiple mechanisms, and conversely, a reduction in intracellular c-di-GMP levels inhibits biofilm formation or triggers dispersal^12^. C-di-GMP is synthesized by diguanylate cyclases (DGCs) with a core GGDEF catalytic domain and is hydrolysed by phosphodiesterases (PDEs) with either EAL or HD-GYP core domains^13^. As such, inhibition of DGCs or stimulation of PDEs is considered a promising way to decrease c-di-GMP levels and interfere with biofilm formation and susceptibility^14^.

However, the c-di-GMP signalling pathway is a highly complex network, where multiple genes encoding DGCs and PDEs collectively contribute to modulating the c-di-GMP concentration in response to a myriad of environmental stimuli^15,16^. Not all of these proteins influence c-di-GMP levels in the same way: some DGCs and PDEs alter the overall level, while others fine-tune local concentrations^16,17^. In addition, the production or hydrolysis of c-di-GMP may occur in a stepwise manner throughout the course of biofilm development, where different DGCs/PDEs function as the predominant regulators in each distinct stage^18^. The plasticity and sophistication of this highly organized pathway hence present a major challenge for the development of effective small molecules that interfere with c-di-GMP signalling.

A number of compounds that inhibit or disperse surface-associated biofilms by inhibiting DGCs, stimulating PDEs, controlling riboswitches, or sequestering c-di-GMP by direct binding have been identified^11,19–21^. However, while such surface-associated biofilms have been extensively studied, more recent evidence demonstrated that biofilms in chronic infections predominantly form suspended (non-attached) aggregates surrounded by host secretions and inflammatory cells^22–26^. While surface-attached biofilms and non-attached aggregates share similar phenotypes^22^, it is currently unclear whether agents targeting surface-attached biofilms can also inhibit non-attached aggregates in more complex in vivo-like conditions. In the present study, we aimed to discover novel DGC inhibitors that reduce auto-aggregation of *P. aeruginosa*, a widespread opportunistic human pathogen associated with many diseases, of which the most well-documented is chronic lung infections in cystic fibrosis (CF) patients. To generate a more in vivo*-*like environment, *P. aeruginosa* aggregates were grown in synthetic cystic fibrosis medium 2 (SCFM2), which is a validated pre-clinical model for studying *P. aeruginosa* gene expression observed in the cystic fibrosis lung^27^. Forty-two *P. aeruginosa* proteins contain GGDEF, EAL/HD-GYP, or dual domains^28,29^, and one of these (WspR) has been the target for all previously identified DGC inhibitors^30–33^. However, we found in this study that deleting *wspR* in *P. aeruginosa* PAO1 did not affect the overall auto-aggregation level in SCFM2. Therefore, we compared the expression levels of 40 genes encoding DGCs and PDEs between aggregated and planktonic cells of 32 *P. aeruginosa* clinical isolates. This led to the identification of SiaD, a previously reported DGC responsible for *P. aeruginosa* auto-aggregation under the challenge of detergent^34,35^, as a key player in aggregation in SCFM2. By in silico screening of 21,495 bioactive compounds against SiaD, echinacoside was identified as a potential inhibitor, and we subsequently showed it reduced c-di-GMP levels, inhibited aggregation in SCFM2, and increased the efficacy of tobramycin against pre-established *P. aeruginosa* aggregates. Furthermore, a synergistic effect of echinacoside and tobramycin was observed towards *P. aeruginosa* PA14 aggregates in a physiologically relevant in vitro model of lung epithelial cells (3-D human A549 cells^36–38^) as well as in vivo (mouse lung infection model^39^). As such, our study not only identified an essential DGC responsible for *P. aeruginosa* auto-aggregation in CF sputum environment suitable for drug development but also discovered a compound with high potential for improving *P. aeruginosa* treatment outcome in future clinical applications.

## Methods

### Strain and culture conditions

Bacterial strains and constructed plasmids in this study are listed in Supplementary Table 2. The incubation time for each strain is listed in Supplementary Table 4. Overnight cultures were grown in Lennox lysogeny broth (LB, Neogen, UK). Bacterial aggregates were grown in SCFM2 prepared as previously described^40^ while corresponding planktonic cultures for cyclic di-GMP ELISA assay and RNA extraction were grown in SCFM2 without salmon sperm DNA and mucin.

### Genetic manipulation in PAO1 and PA14

In‐frame, seamless gene deletion on the PAO1 chromosome and complementary strains were constructed as previously described^41^. Primer pairs are listed in Supplementary Table 6. To generate GFP-tagged PA14, pBK-miniTn7-gfp2 plasmid in *E. coli* DH5α was conjugated into PA14 through four-parental mating as previously described^41^. Colonies grown on PIA containing 100 μg/mL gentamicin were subjected to the fluorescent microscope for confirmation. The construction of chromosomally integrated *siaA* promoter::*lacZ* transcriptional fusion in PAO1 (PAO1 *PsiaA::lacZ*) was performed by fusing the 500 bp region upstream of *siaA* to the promoterless pUC18-mini-Tn7T-Gm-*lacZ* by Gibson assembly. pUC18-mini-Tn7T-Gm-*PsiaA*-*lacZ* was transformed into PAO1 together with pTNS2 helper plasmid, and the successful fusion at the *att::Tn7* site downstream of *glmS* was selected on LB agar containing 75 µg/mL gentamicin and confirmed by PCR. Primer pairs are listed in Supplementary Table 6.

### Whole genome sequencing, RNA extraction and RT-qPCR

Genomic DNA from different isolates was obtained from pelleted overnight cultures using the Wizard Genomic DNA purification kit (Promega) following the manufacturer’s instructions. The quality of extracted DNA was measured using BioDrop μLITE (BioDrop, Cambridge, UK) before library preparation (350 bp) and Illumina sequencing (NovaSeq PE150, Novogene, UK). Contigs were assembled using the QIAGEN CLC genomic workbench. Accession numbers are listed in Supplementary Table 2. Each c-di-GMP metabolism-related gene in each strain was identified via BLASTN (Supplementary Table 3).

For total RNA extraction, overnight cultures of different strains were washed twice in 0.9% NaCl before inoculated into SCFM2 (for aggregate culture) and SCFM2 w/o DNA and mucin (for planktonic culture) at a final concentration of 1 × 10^7^ CFU/mL. Aggregates were grown statically in U-bottom 96-well plates (200 µL each well) in a microaerophilic incubator (3% O_2_, 5% CO_2_) at 37 °C. Planktonic cells were grown in 15 mL falcon tubes (3 mL each) with limited access to O_2_ (lid screwed tight) at 37 °C with vigorous shaking (250 rpm). After the desired incubation time for different strains (Supplementary Table 4), 1200 µL combined bacterial aggregates or 600 µL planktonic cells were mixed 1:1 with RNAlater (Invitrogen, Thermo Fisher). Samples were then pelleted at 5000×*g* at 4 °C for 15 min and re-suspended in 200 µL of freshly prepared 25 mg/mL lysozyme (Thermo Fisher) dissolved in 1×Tris–EDTA buffer (Fisher). The total RNA in each sample was extracted using QIAGEN RNeasy minikit and RNase-free DNase set following the manufacturer’s instructions. The residual gDNA in each sample was removed by TURBO DNA-free™ Kit (Invitrogen, Thermo Fisher) following the manufacturer’s instructions. The quality and quantity of RNA in each sample were measured by BioDrop μLITE.

Freshly extracted RNA (500 ng per 20 µL reaction) was transcribed into cDNA using a High-Capacity cDNA Reverse Transcription Kit (Applied Biosystems, Thermo Fisher). Such cDNA samples were applied as templates for RT-qPCR using 2× GoTaq® qPCR Master Mix (Promega). The reactions were performed on the CFX96^TM^ system (BIO-RAD) with the following PCR programme: 95 °C for 3 min; 40 cycles of 95 °C for 15 s, Tm for 30 s and 72 °C for 15 s; plate read and melt curve generation. RT-qPCR primers for each gene were designed using Clone Manager 8, and the sequence of each primer was 100% conserved in all tested strains (Supplementary Table 6). The specificity of each primer pair was tested by PCR, and the optimal annealing temperature was tested via thermal gradient RT-qPCR using PAO1 cDNA as the template. The stability of 7 frequently used housekeeping genes (*ampC*, *oprD*, *rpsL*, *fabD*, *rpoS*, *gyrA*, *proC*) in *P. aeruginosa* was tested using RefFinder^42^, which integrates the calculation from 4 major computational programmes (geNorm, Normfinder, BestKeeper, and the comparative *Δ*-Ct method). An appropriate weight to each gene was assigned, and the geometric mean of their weights was calculated for the comprehensive ranking (Supplementary RT-qPCR dataset; Supplementary Fig. 1e). The relative transcript levels of target genes were then quantified by being normalized against *proC* showing the highest stability. Heatmap was generated by an online Web tool: https://biit.cs.ut.ee/clustvis/^43^. Briefly, the mean fold-change value (aggregate vs. planktonic) of each gene in each strain was imported into Clustvis. Data was pre-processed without transformation or row centring, but with unit variance scaling for each row. Such scaling for each data point in the heatmap was calculated as below to achieve a standard deviation of 1 for each gene across all strains.

$$\frac{{{{fold}}\; {{change}}\; {{value}}\; {{of}}\; {{gene}}\, X\,{{in}}\; {{strain}}\,Y}}{{{{standard}}\; {{deviation}}\; {{of}}\; {{the}}\; {{fold}}\; {{change}}\; {{values}}\; {{of}}\; {{gene}}\,X\,{{across}}\; {{all}}\; {{strains}}}}$$

After such data pre-processing, the heatmap was then generated to show imputed values for missing value estimation, and rows (genes) were clustered using correlation distance and average linkage, where order branches of the clustering tree of rows were arranged to show higher mean values first. In contrast, columns (strains) were not clustered.

### β-galactosidase assays

To compare the expression level of *siaA* in aggregates and planktonic cells grown in SCFM2 and SCFM2-mucin-DNA, respectively, PAO1 WT (*lacZ*^-^ negative control) and PAO1 *PsiaA*::*lacZ* were cultured under identical conditions as those cultured for RNA extraction. After 6-h incubation, bacterial cells were immediately transferred on ice to arrest growth. 1.6 mL PAO1 *PsiaA::lacZ* aggregates samples were pelleted at 5000×*g* at 4 °C for 15 min and re-suspended in 200 µL SCFM2-mucin-DNA. 100 µL concentrated aggregate samples or 100 µL planktonic cells were incubated with 900 μL lysis buffer (60 mM Na_2_HPO_4_·7H_2_O, 40 mM NaH_2_PO_4_·H_2_O, 10 mM KCl, 1 mM MgSO_4_, 7.7 mM β-mercaptoethanol, 0.001% SDS) and 20 μL chloroform at 28 °C for >10 min until cells lysed. 200 μL of 4 mg/mL ONPG was added, and samples were monitored until the substrate had turned yellow. 500 μL of 1 M Na_2_CO_3_ was added to stop the reaction, and the absorbance was measured at 420 and 550 nm using a FLUOstar plate reader (BMG). The remaining samples were subjected to CFU counting and converted to OD_600nm_ values. Miller Units were calculated as previously described^44^.

### Virtual screening and molecular docking

The software used for virtual screening and 3-D mapping were Schrödinger Maestro 11.4 and PyMOL, respectively. For protein structure preparation, the crystal structure of SiaD was obtained from http://www.rcsb.org/ (PDB ID: 7E6G). 21,495 bioactive compounds, including natural products, enzyme inhibitors, receptor ligands, and drugs from the MCE Bioactive Compound Library (MedChemExpress, HY-L001V), were prepared using the LigPrep module. The Schrödinger software used for virtual screening here uses the Glide module, which applies a series of hierarchical filters to search for possible locations of ligands in the receptor active site region. The shape and properties of the receptor are represented on a grid by different sets of fields, which provides accurate scoring of the ligand pose (i.e. specification of the ligand, including position and orientation relative to the receptor, core conformation, and rotamer-group conformations). These pre-processing steps were then followed by docking to produce sets of initial and refined ligand conformations, and the software generates GlideScore based on a modified version of the ChemScore function described previously to predict binding affinity and rank-order ligands in database screens^45^. The combination of GlideScore, the ligand−receptor molecular mechanics interaction energy, and the ligand strain energy is used to select the correctly docked pose. This composite scoring function generates a negative value as a docking score. The lower this docking score is, the stronger the binding ability of the protein to the small molecule compound. A docking score of −10 points theoretically predicts a strong potential binding ability. Compounds were initially docked by the Glide high-throughput virtual screening (HTVS) mode. The top 15% ranked compounds were chosen and redocked by the Glide standard precision (SP) scoring mode. Then the top 15% ranked compounds from this selected group were subjected to another round of docking by the extra precision (XP) scoring mode.

### Evaluation of the susceptibility to antibiotics and selected compounds

The Minimum inhibitory concentration (MIC) values of tobramycin (MedChemExpress), ciprofloxacin hydrochloride monohydrate (MedChemExpress), ceftazidime (MedChemExpress) and meropenem (Fresenius Kabi, Belgium) were determined in Mueller-Hinton broth (MHB, Neogen, UK) using EUCAST microdilution method. The tested concentration range of each antibiotic was 0.0625–512 µg/mL. Pre-established aggregates of different *P. aeruginosa* strains were grown in SCFM2 in U-shape 96-well plates in a microaerophilic incubator after desired periods (Supplementary Table 4). The efficacy of each antibiotic against pre-established aggregates was determined within the working concentration range of 4 × MIC–512 µg/mL after 18-h treatment. Treated and untreated samples were collected into 2 mL reinforced tubes and subjected to bead ruptor 24 elite (OMNI, USA) for aggregate disruption (4000 rpm, 2 min), and CFU numbers were determined by plating. Concentrations of different antibiotics where at least 1-log reduction of CFU can be observed compared to untreated samples but did not completely eradicate aggregates were used for further tests.

Different concentrations of echinacoside were applied either alone or with different antibiotics at the abovementioned concentrations. Echinacoside was dissolved in H_2_O at a concentration of 20 mM as a stock solution. The bactericidal effect of compounds alone and the effect of combination treatments were determined by plating and CFU count, as abovementioned. For testing the inhibitory effect of echinacoside, different concentrations of echinacoside were applied together with the initial inoculum of PAO1 in SCFM2 (1 × 10^5^ CFU/mL). After 6 h, 16 µg/mL tobramycin was added to the pre-established aggregates with existing echinacoside treatments for a further 18-h incubation prior to plating and CFU enumeration. To evaluate whether echinacoside serves as a nutrient source for *P. aeruginosa* cell growth, 1 × 10^5^ CFU/mL PAO1 was inoculated into standard M9 medium without glucose (64 g/L Na_2_HPO_4_·7H_2_O, 15 g/L KH_2_PO_4_, 2.5 g/L NaCl, 5 g/L NH_4_Cl, 0.1 mM CaCl_2_, and 2 mM MgSO_4_), M9 minimal medium without glucose but with 25, 50, 100, 200, and 400 µM echinacoside (the same concentration range used in combination treatment assay), and M9 minimal medium (with 4 g/L glucose) in 96-well plates (200 µL per well). The OD_600nm_ measurement was performed in a FLUOstar plate reader (BMG) every 30 min over 24-h incubation at 37 °C.

### Quantification of intracellular c-di-GMP levels

Intracellular c-di-GMP quantification for aggregates and planktonic cultures was accomplished by c-di-GMP ELISA kit (Cayman, USA). To compare the difference between aggregated and planktonic samples, bacteria were cultured under identical conditions as those cultured for RNA extraction. To assess the effect of echinacoside on c-di-GMP, different concentrations of the compound were added together with bacteria diluted in SCFM2 (1 × 10^5^ or 1 × 10^7^ CFU/mL). After desired incubation, 3000 µL (initial inoculum 1 × 10^5^ CFU/mL) or 1200 µL (initial inoculum 1 × 10^7^ CFU/mL) aggregate samples and 600 µL planktonic samples were collected and immediately transferred to pre-chilled centrifuge tubes kept on ice. 100 µL of each sample was spared for CFU determination. Samples were centrifuged at 5000×*g* for 15 min at 4 °C, and the supernatant was discarded. Pellets were then completely dissolved in 200 µL B-PER™ bacterial protein extraction reagent (Thermo Fisher) for 15 min at room temperature prior to centrifugation (15,000×*g*, 10 min). Supernatants from aggregate and planktonic samples were 3-fold and 6-fold diluted into B-PER solution, respectively, for ELISA assay, and 2-fold diluted into MQ H_2_O to measure total protein concentration. Diluted samples were subjected to c-di-GMP ELISA kit and Pierce™ 660 nm protein assay reagent (Thermo Fisher) following manufacturer’s instructions. 2 mg/mL bovine serum albumin (BSA, Thermo Fisher) was used to determine standard curves for protein quantification. Total c-di-GMP was then normalized to CFU or total protein.

### 3-D lung epithelial model infection assay and cytotoxicity assay

The 3-D in vivo-like lung model was established from the human alveolar epithelial cell line A549 (ATCC CCL-185) using GTSF-2 medium (HyClone, Logan, UT, USA) as previously described^37,38^. A549 cells were first cultured in tissue culture flasks and then transferred to an optimized suspension culture bioreactor termed the rotating wall vessel (RWV) containing porous extracellular matrix (ECM)-coated microcarrier beads and culture medium. As RWVs rotate, both the wall and the fluid mass rotate at the same angular rate, creating a constant freefall of the cells through the culture medium. As cells are maintained in a gentle fluid orbit, they attach to the microcarrier beads and each other to form the connections required for a more “tissue-like” phenotype^37^. The 3-D model of the A549 cell line reflects key phenotypic characteristics of in vivo lung epithelial cells, including barrier function, apical and basolateral polarity, and mimics aspects of the *P. aeruginosa* in vivo infection process such as the formation of biofilms^37,38,46,47^. On the day of infection, an equal number of microcarrier beads containing 3-D A549 cells were transferred into each well in flat-bottom 24 well plates to achieve ⁓2.5 × 10^5^ cells/well. Mid-log phase GFP-tagged PA14 (for microscopic analysis) or PA14 (for cytotoxicity assay) bacterial suspensions grown in LB were washed twice in GTSF-2 medium without FBS and inoculated into each well containing 3-D A549 cells at a multiplicity of infection (MOI) of 100:1. After 4-h static co-culture in a microaerophilic incubator at 37 °C to allow for bacterial adherence, cells were washed twice with GTSF-2 without FBS to discard planktonic or loosely attached PA14. PA14-infected 3-D A549 cells were treated with tobramycin, echinacoside, or both together for 18 h in a microaerophilic incubator statically. The viability of A549 cells with and without different treatments was quantified using ‘intracellular’ lactate dehydrogenase (LDH) assay as previously described^48^.

### Murine lung infection assay and evaluation

All animal experiments were approved by the Laboratory Animal Welfare and Ethics Committee of Shanghai Pudong Hospital (No. 20240413-001). Twenty-five 12-week-old female BALB/c mice were randomly grouped into 5 groups (5 each group based on G*Power calculation) and managed according to the guidelines provided by the Shanghai Pudong Hospital ethics committee. Mouse rooms were maintained at 25 °C, with 70% humidity and 12 hr light-dark cycle. Investigators conducting the animal experiments were blinded to the specific treatment for each group of mice. To mimic chronic infections in airways, overnight cultures of PA14 were immobilized in LB agar beads, as previously described, to reach a concentration of ~1.2 × 10^9^ CFU/mL^39^. 40 µL of such freshly encapsulated *P. aeruginosa* agar beads were administered to each anaesthetised mouse by intratracheal instillation as reported before^49^, enabling a final inoculation of ~4.8 × 10^7^ CFU/mouse. 40 µL sterile empty agar beads were used as a negative control. Upon regaining consciousness, mice had unlimited access to feed and water. After 22-h infection, a total volume of 30 µL solutions containing sterile H_2_O and 7.2 µg tobramycin and/or 5.2 µg echinacoside, or just 30 µL sterile H_2_O, was applied intratracheally to different groups of anaesthetised mice. After 8 h, mice were sacrificed by cervical dislocation, and the bacterial burden in each whole lung was determined by homogenization in phosphate-buffered saline (PBS) and plate counting.

### Microscopic analysis

For observing bacterial aggregates, overnight cultures of PAO1 WT and mutants were washed twice in 0.9% NaCl and diluted into SCFM2. To compare the size of aggregates formed by different isogenic mutants, diluted bacteria suspensions at a final concentration of 1 × 10^5^ CFU/mL in SCFM2 were inoculated into flat-bottom 48 well plates (1 mL per well) and incubated in a microaerophilic incubator statically for 24 h. To assess the effect of selected compounds on auto-aggregation, different concentrations of compounds were added together with PAO1 WT diluted samples (1 × 10^5^ CFU/mL) and incubated statically for 6 h. To observe aggregates (grown statically) and planktonic cells (grown with 250 rpm shaking), overnight cultures were diluted to a final concentration of 1 × 10^7^ CFU/mL in SCFM2 or SCFM2 without mucin and DNA. After incubation, samples were harvested carefully with wide-orifice tips (Finntip, Thermo Fisher) from the middle layer of bacteria suspension in each well (aggregate)/each tube (planktonic) and 4-fold (aggregate)/12-fold (planktonic) diluted into SCFM2 without DNA and mucin. 200 µL such diluted samples were inoculated into each well in an uncoated Ibidi 96-well plate (Ibidi, USA) and subjected to EVOS FL Auto microscope (Thermo Fisher). Micrographs were obtained using ×20 objective with transmitted light. The gap between each z-stack was 0.488 µm. The size of each aggregate in image stacks was quantified by Fiji as previously described^41^. For observing the overall morphology/integrity of 3-D lung epithelial cells and attached GFP-tagged PA14, micrographs were obtained using ×10 objective with both transmitted light and GFP fluorescent light cube (470 nm excitation, 525 nm emission).

### Statistical analyses

Quantitative data sets were analysed by calculating the mean and standard deviation of independent biological replicates for each experiment. The figure legends provide detailed information about replicates and statistical analyses for each experiment. All statistical analyses were performed in Graphpad Prism 9.

## Results

### SiaD (PA0169) is an essential DGC responsible for *P. aeruginosa* auto-aggregation in SCFM2

**Fig. 1:**
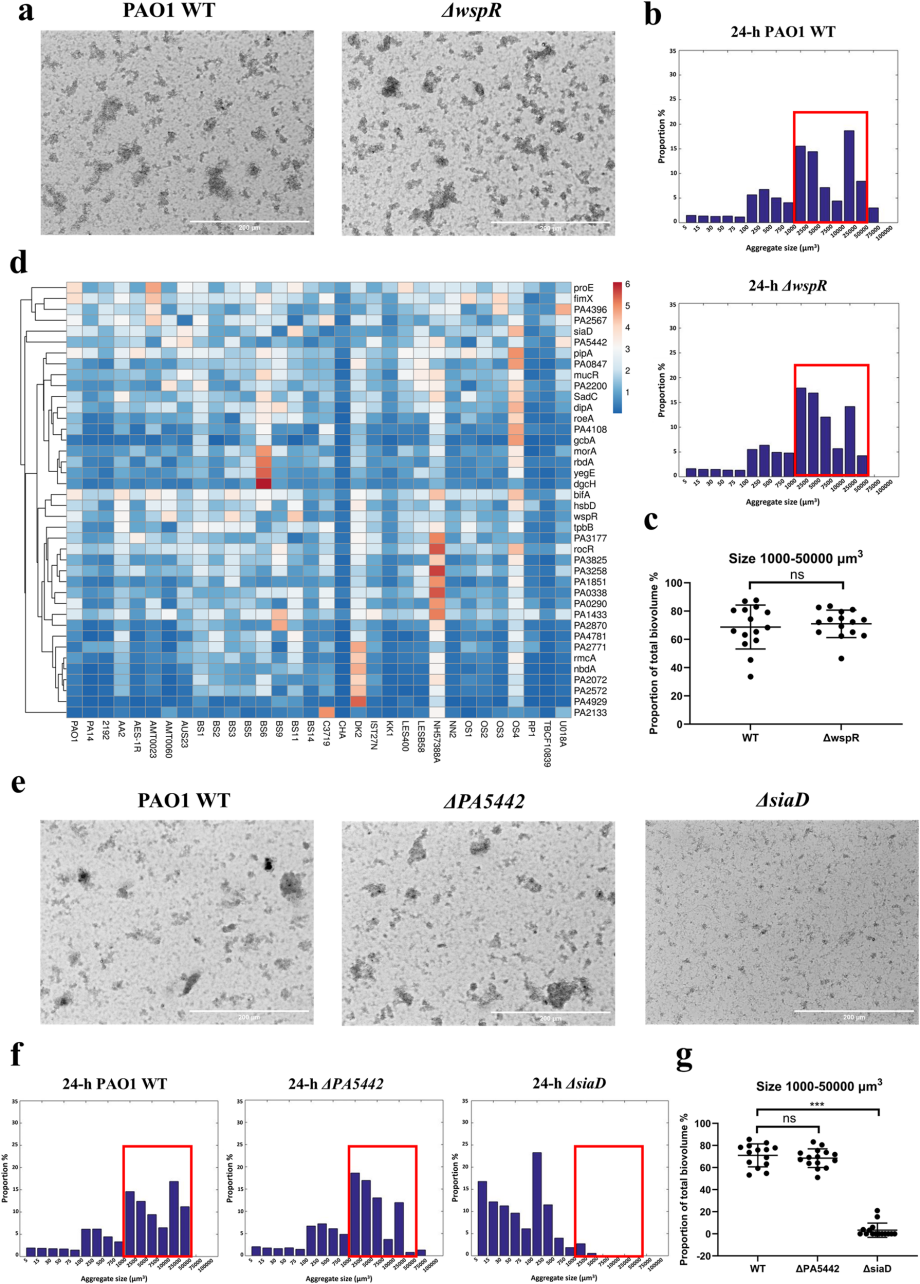
SiaD is essential for *P. aeruginosa* aggregation in SCFM2. **a** Representative micrographs of *P. aeruginosa* PAO1 WT and *ΔwspR* aggregates grown in SCFM2 for 24 h. Scale bar = 200 µm. **b** Distribution of the size of PAO1 WT and *ΔwspR* aggregates grown in SCFM2 for 24 h. The biovolume (µm^3^) of single cells and aggregates were grouped into 17 categories. The biovolume of all aggregates belonging to each size category was calculated, and the proportion of this biovolume of each category in the total biovolume was calculated. Histograms represent the mean proportion value of all samples collected from 3 individual experiments (*n* = 3), where micrographs were obtained from at least 3 random locations in each sample. Numerical data and standard deviations are shown in Supplementary Table 1. **c** Proportion of aggregates with sizes ranging from 1000 to 50,000 μm^3^ in total biovolume. ***p < 0.001 (Student’s *t*-test). **d** Heat map showing the relative expression level (fold changes) of genes within the *P. aeruginosa* c-di-GMP network (*y*-axis) in aggregated cells *versus* planktonic cells of 32 strains (*x*-axis). Unit variance scaling is applied to rows. Imputation is used for missing value estimation. Rows are clustered using correlation distance and average linkage. Tree for rows were ordered where genes with higher mean values were shown first. **e** Representative micrographs of PAO1 WT, *ΔPA5442*, and *ΔsiaD* aggregates grown in SCFM2 for 24 h. Scale bar = 200 µm. **f** Distribution of the size of PAO1 WT, *ΔPA5442* and *ΔsiaD* aggregates grown in SCFM2 for 24 h. Histograms represent the mean proportion value of all samples collected from 3 individual experiments (*n* = 3), where micrographs were obtained from at least 3 random locations in each sample. Numerical data of mean proportions and standard deviation are shown in Supplementary Table 1. **g** Proportion of aggregates with sizes ranging from 1000 to 50,000 μm^3^ in total biovolume. ***p < 0.001; ns, not significant (Student’s *t*-test for comparison between two groups; One-Way ANOVA with post-hoc Dunnett’s tests for multiple comparisons between 2 groups when datasets contain 3 or more groups).

*P. aeruginosa* WspR is a well-characterized active DGC associated with c-di-GMP production, surface sensing and cell envelope stress, biofilm formation, and motility^50–53^. However, its role in the formation of non-surface attached *P. aeruginosa* aggregates in clinically relevant models is unclear. We observed that after 24-h incubation, the lack of WspR did not impact the auto-aggregation of *P. aeruginosa* PAO1 in SCFM2 (Fig. 1a). The sizes of aggregates were comparable between WT and *ΔwspR*, with aggregates measuring between 1000 and 50,000 μm^3^ constituting 68.72 ± 15.54% and 71.02 ± 9.73% of total biovolume in WT and *ΔwspR* cultures, respectively (Fig. 1b, c, Supplementary Table 1). Consistently, the late-exponential WT and *ΔwspR* cells formed aggregates with similar sizes after 6-h incubation (Supplementary Fig. 1a and b). Subsequently, we evaluated the expression levels of 40 genes encoding proteins containing GGDEF, EAL/HD-GYP, and dual domains that are involved in c-di-GMP metabolism in 32 *P. aeruginosa* clinical strains (Supplementary Table 2). *arr* and *pvrR* were excluded due to their absence in multiple strains (Supplementary Table 3). We compared gene expression levels between aggregates grown in SCFM2 and planktonic cells grown in SCFM2 without DNA and mucin (mucin and DNA support auto-aggregation more as physical rather than nutritional factors^54–56^) (Supplementary Fig. 1c and d). As the growth phase significantly influences the expression patterns of c-di-GMP-related genes^57^, we cultivated different strains for different periods of time in order for them to reach the same growth phase (i.e. late log-phase) (Supplementary Table 4). The growth states of planktonic cells and aggregates were determined by OD_600nm_ measurement and CFU counting, respectively (Supplementary Fig. 1f). Using RT-qPCR we could not identify genes for which the fold change in expression between aggregates and planktonic cells was substantially different from all other genes among the majority of strains (fold-change values of most genes ranged from ~0.2 to ~2; with a few exceptions in strains DK2, NH57388A, OS4, and BS6) (Fig. 1d, Supplementary RT-qPCR dataset). This finding was consistent with results from our ELISA-based intracellular c-di-GMP quantification assay (Supplementary Fig. 2a), where only 9 out of 32 strains showed a lower intracellular c-di-GMP level in planktonic cells compared to their aggregated counterparts, and the decrease was not as substantial (~3-fold) as reported previously^58,59^. However, for genes *proE*, *fimX, PA4396*, *PA2567*, *siaD* and *PA5442* the overall expression fold changes (aggregates *versus* planktonic cells) in different strains were higher than those of other genes, and these genes occupy distinct positions in the heatmap (Fig. 1d). PA4396 is a degenerated DGC^60^; PA2567 is an EAL-containing PDE^61^; ProE harbours a degenerate GGDEF domain and exhibits PDE activity^62^; and FimX contains GGDEF and EAL domains that are both inactive^63^. SiaD was shown to be an active DGC involved in *P. aeruginosa* auto-aggregation in response to environmental stress^34,35,64^ and PA5442 was predicted to contain an active DGC domain^65^. To assess the role of these two proteins in auto-aggregation in SCFM2, isogenic mutants *ΔsiaD* and *ΔPA5442* were constructed in PAO1. Deleting *PA5442* did not significantly affect the aggregation level, as 71.06 ± 10.37% and 68.54 ± 8.45% of total biovolume were constituted by aggregates with sizes ranging from 1000 to 50,000 μm^3^ in WT and *ΔPA5442* cultures after 24-h incubation, respectively. In contrast, the deletion of *siaD* substantially reduced the size of aggregates, with aggregates larger than 1000 μm^3^ only making up 3.31 ± 6.42% of the total biovolume (Fig. 1e–g, Supplementary Table 1). Similarly, late-exponential WT and *ΔPA5442* cells formed aggregates with comparable sizes, while *ΔsiaD* cells formed much smaller aggregates (Supplementary Fig. 1a and b). Complementing *ΔsiaD* with *siaD* restored the aggregative behaviour (Supplementary Fig. 3 and Supplementary Table 1). Therefore, we concluded that *siaD* is essential for aggregate formation in SCFM2, and SiaD was selected for further studies.

### Echinacoside reduces both auto-aggregation and intracellular c-di-GMP levels of PAO1 in SCFM2

**Fig. 2:**
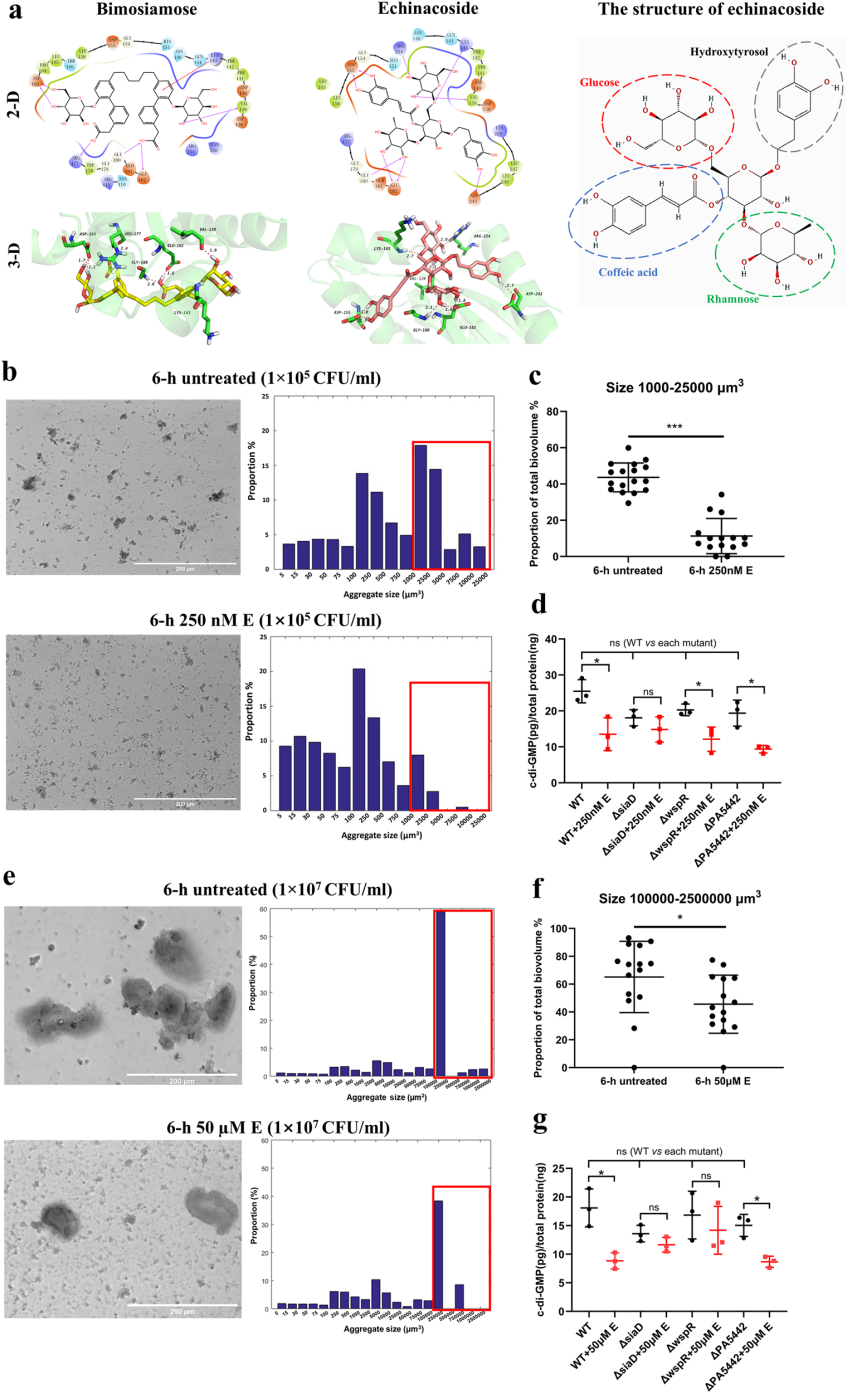
Echinacoside reduces *P. aeruginosa* aggregate sizes and intracellular c-di-GMP levels. **a** Interaction between two compounds with the lowest in silico docking score and SiaD. Chains B/C/D/E/F, water molecules, magnesium ions and the missing hydrogen atoms of SiaD were deleted. In the 3-D illustration (lower panel), the carbon skeleton of SiaD is displayed in green, N atoms in blue, O atoms in red, H atoms in white, and hydrogen bonds as red dashed lines. In corresponding images, bimosiamose and echinacoside were displayed as light yellow and dark pink sticks, respectively. The distances of hydrogen bonds are also shown. The isolated depiction of the structure of echinacoside was adapted from W. Wang et al.^66^. **b** Representative micrographs of *P. aeruginosa* PAO1 aggregates (initial inoculum 1×10^5^ CFU/mL) grown in SCFM2 with or without 250 nM echinacoside for 6 h (Scale bar = 200 µm) and the corresponding distribution of the size of aggregates. The biovolume (µm^3^) of aggregates were grouped into 14 categories. Histograms represent the mean proportion value of all samples collected from 3 individual experiments (*n* = 3), where micrographs were obtained from at least 3 random locations in each sample. **c** Proportion of aggregates in **b** with sizes ranging from 1000 to 25000 μm^3^ in total biovolume. **d** Intracellular c-di-GMP levels in *P. aeruginosa* PAO1 WT, *ΔsiaD, ΔwspR*, and *ΔPA5442* aggregates (initial inoculum 1 × 10^5^ CFU/mL) grown in SCFM2 for 6 h with or without 250 nM echinacoside treatment normalized to total protein concentration (*n* = 3 with two technical replicates each; error bar denotes SD). **e** Representative micrographs of *P. aeruginosa* PAO1 aggregates (initial inoculum 1 × 10^7^ CFU/mL) grown in SCFM2 with or without 50 µM echinacoside for 6 h (Scale bar = 200 µm) and the corresponding distribution of the size of aggregates. The biovolume (µm^3^) of aggregates were grouped into 20 categories. Histograms represent the mean proportion value of all samples collected from 3 individual experiments (*n* = 3), where micrographs were obtained from at least 3 random locations in each sample. **f** Proportion of aggregates in **e** with sizes ranging from 100,000 to 2,500,000 μm^3^ in total biovolume. **g** Intracellular c-di-GMP levels in *P. aeruginosa* PAO1 WT, *ΔsiaD, ΔwspR*, and *ΔPA5442* aggregates (initial inoculum 1 × 10^7^ CFU/mL) grown in SCFM2 for 6 h with or without 50 µM echinacoside treatment normalized to total protein concentration (*n* = 3 with two technical replicates each; error bar denotes SD). ***p < 0.001; *p < 0.05; ns not significant (Student’s *t*-test for comparison between two groups; One-Way ANOVA with post-hoc Dunnett’s tests for multiple comparisons between 2 groups when datasets contain 3 or more groups).

The structure of SiaD deposited in PDB (code 7E6G) was used as the template for virtually docking 21,495 compounds with known bioactivities into the conserved GTP substrate-binding site (GGDEF domain, A-site). The docking score refers to the binding affinity between these compounds and the coordination pocket of SiaD, with lower docking scores correlating with higher binding affinity and generally a score lower than -10 (i.e. <−10 kcal/mol) suggests strong binding. Results showed that bimosiamose and echinacoside exhibited the highest binding affinity among 21,495 compounds tested, with docking scores of −10.260 and −10.259, respectively (Fig. 2a, Supplementary Excel top 200 compounds identified by virtual docking). Bimosiamose can form six hydrogen bonds with Asp103, Arg177, Gly180, Glu182, and Val139, and the benzene ring can form one cation-π interaction with Lys143. Multiple hydroxyl groups of echinacoside can form nine hydrogen bonds with Asp155, Glu182, Gly180, Asp243, Val139, Lys143, and Arg254 (Fig. 2a, upper panel). While neither of these two compounds showed a bactericidal effect at concentrations tested (Supplementary Fig. 4a), only echinacoside reduced the aggregation level of PAO1 in SCFM2. Echinacoside ([(2R,3R,4R,5R,6R)-6-[2-(3,4-dihydroxyphenyl)ethoxy]-5-hydroxy-2-[[(2R,3R,4S,5S,6R)-3,4,5-trihydroxy-6-(hydroxymethyl)-oxan-2-yl]oxymethyl]-4-[(2S,3R,4R,5R,6S)-3,4,5-trihydroxy-6-methyloxan-2-yl]oxyoxan-3-yl](E)-3-(3,4-dihydroxyphenyl)prop-2-enoate; C_35_H_46_O_20_) is a water-soluble phenylethanoid glycoside extracted as one of the primary active ingredients from the roots of plant *Echinacea angustifolia*. Currently, it is classified as an experimental stage small molecule based on its diverse biological activities shown in human cells and different animal models, including antioxidant, neuroprotection, anticancer activity, bone regeneration, and hepatoprotective activity^66^. When 1 × 10^5^ CFU/mL PAO1 was incubated with 250 nM echinacoside, after 6 h, only 11.28 ± 9.68% of bacterial cells grew into aggregates larger than 1000 μm^3^. In contrast, 43.61 ± 7.94% of the total biovolume of untreated cells was composed of aggregates larger than 1000 μm^3^ (Fig. 2b, c, Supplementary Table 1). A similar aggregate-inhibiting effect of echinacoside could also be observed when a higher concentration of PAO1 (1 × 10^7^ CFU/mL) was incubated with a higher concentration of the compound (50 µM) after 6 h, where untreated and treated groups contained 65.15 ± 25.61% and 45.60 ± 20.89% bacterial cells that grew into aggregates larger than 100,000 μm^3^, respectively (Fig. 2e, f, Supplementary Table 1). However, this inhibitory effect was less prominent against the higher inoculum (1 × 10^7^ CFU/mL) than when the inoculum was 1 × 10^5^ CFU/mL. It is postulated that in SCFM2 containing high concentrations of polymers (DNA and mucin), the addition of a higher concentration of bacterial cells may result in rapid auto-aggregation probably driven by entropic forces that do not require biofilm formation functions^67^. This depletion aggregation may have, to some extent, hindered the observation that would have been acquired just from a reduction in intracellular c-di-GMP level. Interestingly, the effect of echinacoside on the aggregation of PAO1 in SCFM2 was concentration-dependent, and the reduction was abolished when echinacoside was applied at concentrations higher than 1 or 100 µM with the initial inoculum size being 1 × 10^5^ or 1 × 10^7^ CFU/mL, respectively (Supplementary Fig. 5a and b and Supplementary Table 1). This concentration-dependent behaviour of echinacoside was not because the compound acted as a carbon nutrient source, as echinacoside did not promote cell growth either in SCFM2 or M9 minimal medium without glucose (Supplementary Fig. 4a and b). While the mechanism underlying this phenomenon requires further investigations, we applied echinacoside at a final concentration of 250 nM or 50 µM to 1 × 10^5^ CFU/mL or 1 × 10^7^ CFU/mL PAO1, *ΔsiaD, ΔwspR*, and *ΔPA5442* cells to evaluate if echinacoside can reduce intracellular c-di-GMP levels. As shown in Fig. 2d, g, echinacoside reduced the c-di-GMP concentration in PAO1 WT and *ΔPA5442* regardless of the initial inoculum, and the effect on *ΔwspR* differed depending on the initial inoculum sizes. In contrast, this reduction was abolished for *ΔsiaD* cultured in SCFM2, suggesting the interaction between SiaD and echinacoside is contributing to the reduced intracellular c-di-GMP levels. The small but statistically significant reduction in c-di-GMP levels can be explained by the technical limitation where planktonic cells were inevitably introduced into aggregate samples. As shown in Figs. 1 and 2, the samples were always a mixture of aggregates and planktonic cells, and it was technically impossible to separate aggregates from planktonic cells without drastically changing c-di-GMP under current conditions. As a result, compared to well-established methods for comparing the intracellular c-di-GMP levels of surface-attached biofilms and planktonic cells^68^, our experimental setup cannot generate data that clearly distinguish c-di-GMP levels in aggregates and planktonic cells, which may have weakened our conclusion. As SCFM2 contains many fluorescent chemical ingredients, another well-established method for evaluating intracellular c-di-GMP levels using the P*cdrA*::*gfp* plasmid^69^ would not produce an accurate conclusion in this assay either.

### Echinacoside potentiates the activity of tobramycin against *P. aeruginosa* aggregates formed by clinical isolates

**Fig. 3:**
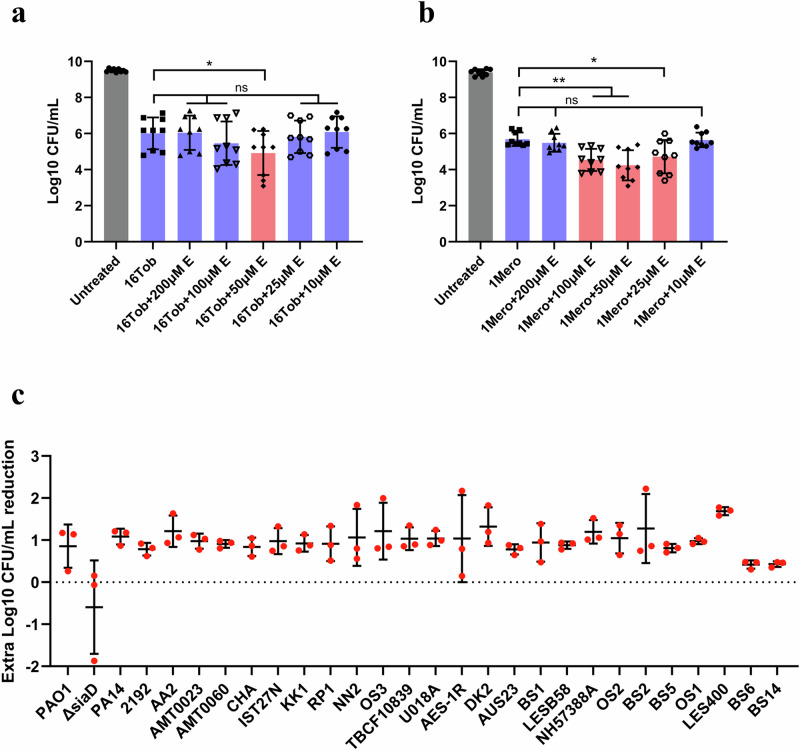
Echinacoside potentiates the activity of tobramycin against *P. aeruginosa* aggregates in SCFM2. **a** and **b** The effect of 16 μg/mL tobramycin (16Tob) (**a**) and 1 μg/mL meropenem (1Mero) (**b**) in combination with different concentrations of echinacoside (10–200 μM E) against pre-established PAO1 aggregates grown in SCFM2 for 6 h. Data are expressed as the mean number of CFU remaining after an additional 18-h incubation (3 independent experiments with 3 technical replicates; error bars indicate standard deviation). Red bars highlighted the successful combination treatment that potentiated the efficacy of antibiotics. *p < 0.05; **p < 0.01 (one-way ANOVA, with post-hoc Dunnett’s tests for multiple comparisons between 2 groups). **c** Extra Log reduction achieved by combination treatment of pre-established aggregates of 27 *P. aeruginosa* strains, compared to treatment with tobramycin alone was shown (3 independent experiments with 3 technical replicates; error bars indicate standard deviation).

As echinacoside inhibited the formation of aggregates, we next assessed whether it could potentiate the activity of frequently used antibiotics. Echinacoside was first applied to pre-established PAO1 aggregates in SCFM2 along with 4 antibiotics belonging to different classes (tobramycin, ciprofloxacin, meropenem, and ceftazidime). Echinacoside enhanced the antimicrobial activity of both tobramycin and meropenem, but not that of ciprofloxacin or ceftazidime (Fig. 3a, b, Supplementary Fig. 6a). Consistent with the ‘golden concentration window’ of echinacoside on reducing PAO1 aggregate sizes as shown in Supplementary Fig. 5c and Supplementary Table 1, its antibiotic-potentiating effect was also concentration-dependent. When this screening was expanded to 7 additional *P. aeruginosa* strains, the potentiating effect was more consistent for tobramycin than for meropenem (for certain strains, the addition of echinacoside even appeared to weaken the bactericidal effect of meropenem; Supplementary Fig. 7). In addition, treating the initial planktonic inoculum with echinacoside for 6 h prior to the addition of tobramycin also promoted the killing (Supplementary Fig. 6b). Therefore, we selected tobramycin for further experiments. Different combinations of echinacoside (25, 50, 100, 200, 400, or 800 µM) and tobramycin (at least two different concentrations leading to ≥3-log reduction in CFU) were tested on pre-established aggregates of 32 *P. aeruginosa* strains grown for different periods in SCFM2 (Supplementary Table 4) to optimize concentration ranges. For 27/32 strains (BS3, BS9, BS11, OS4, and C3719 being the exceptions), the addition of echinacoside led to an extra 0.43 to 1.68 log-fold reduction compared to treatment with tobramycin alone (Fig. 3c, Supplementary Fig. 8). It is currently unclear why echinacoside did not potentiate tobramycin activity against strains BS3, BS9, BS11, OS4, and C3719. BS3, BS9, and C3719 are resistant to tobramycin (Supplementary Table 5; EUCAST breakpoint for resistance is 2 µg/mL), but echinacoside did potentiate tobramycin activity against other tobramycin-resistant strains (e.g. BS5 and DK2). Likewise, BS11 and OS4 are highly mucoid, but echinacoside did potentiate tobramycin activity against other mucoid strains (e.g. BS1 and BS5).

### Echinacoside potentiates the efficacy of tobramycin against *P. aeruginosa*-infected 3-D human alveolar epithelial cells and murine lungs

**Fig. 4:**
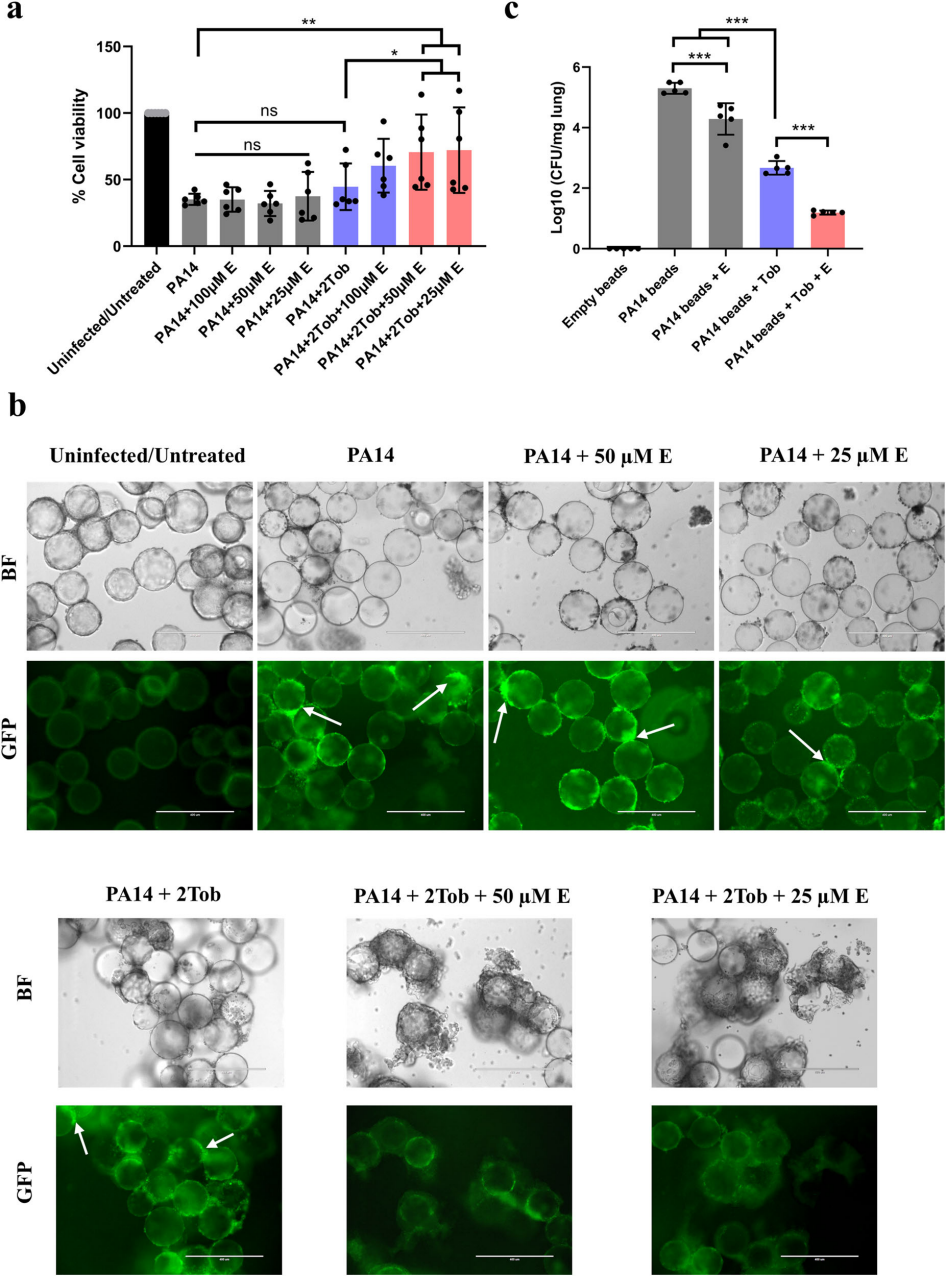
The synergistic effect of echinacoside and tobramycin against *P. aeruginosa* aggregates in human cells and murine lungs. **a** Viability of PA14-infected 3-D A549 cells (as measured with an LDH assay) exposed to different concentrations (25, 50, and 100 μM) of echinacoside, 2 μg/mL tobramycin (2Tob) or the echinacoside/tobramycin combination treatments normalized to untreated/uninfected groups. Red bars highlighted the successful combination treatment that potentiated the efficacy of tobramycin and elevated cell viability. (6 independent experiments with four technical replicates; error bars indicate standard deviation). *p < 0.05; **p < 0.01 (one-way ANOVA, with post-hoc Dunnett’s tests for multiple comparisons between 2 groups). **b** Representative transmission light micrographs (upper panel) of 3-D A549 cells attached to/detached from collagen-treated carrier beads and fluorescent micrographs (lower panel) of GFP-tagged PA14 attached to A549 cells. Some typical PA14 aggregates attached to A549 cells with strong fluorescent signals are highlighted with white arrows. Scale bar = 400 μm. **c** The bacterial load in each uninfected lung with empty agar beads, PA14-infected lung, or PA14-infected lung after exposure to 7.2 µg/mouse tobramycin (Tob), 5.2 µg/mouse echinacoside, or the echinacoside/tobramycin combination treatments. The red bar highlighted the successful combination treatment that potentiated the efficacy of tobramycin. Each group contained 5 mice, and 3 technical replicates were performed for CFU counting. Error bars indicate standard deviation, ***p < 0.001 (one-way ANOVA, with post-hoc Dunnett’s tests for multiple comparisons between 2 groups).

We subsequently evaluated if the synergistic effect could also be observed in a 3-D model of human alveolar epithelial (A549) cells that recapitulates some key aspects of in vivo tissue^37,38^. The widely used reference strain PA14 was chosen for infection assays due to its higher virulence compared to PAO1 and well-documented cytotoxicity against A549 cells^70–73^. After 4 h co-culture of 3-D A549 cells and PA14, aggregates were already found attached to the epithelial cells (Supplementary Fig. 9a). 18-h echinacoside treatment was not cytotoxic to 3-D A549 cells at concentrations ranging from 25 to 400 µM (Supplementary Fig. 9b and c). We subsequently assessed the viability of PA14-infected 3-D A549 cells after different treatments. While the survival of PA14-infected cells treated with echinacoside or 2 μg/mL tobramycin alone was comparable to that observed without treatment, cells treated with 25 or 50 µM echinacoside in combination with tobramycin showed significantly higher survival (Fig. 4a). This was confirmed microscopically: when infected A549 cells were treated with both tobramycin and echinacoside, more cells remained attached to beads than when cells were treated with tobramycin or echinacoside alone (Fig. 4b). In conclusion, at the concentrations tested (µM range) echinacoside was not cytotoxic, and a synergistic effect of echinacoside and tobramycin was observed against *P. aeruginosa* PA14 aggregates formed on 3-D A549 cells.

We subsequently tested whether echinacoside can also potentiate the activity of tobramycin in vivo. Echinacoside was previously shown to be non-toxic to mice^74–76^. PA14 cells embedded in agar beads were intratracheally instilled into 12-week-old female BALB/c mice, with an initial challenge of ~4.8 × 10^7^ CFU/mouse to allow for the formation of aggregates in the lungs. After 22 h, different treatments were administered for 8 h, followed by the determination of the number of surviving *P. aeruginosa* cells. Mock treatment (water) resulted in a bacterial titre of around 2.2 × 10^5^ CFU/mg lung after 30 h. Treatment with tobramycin (7.2 µg/mouse) alone for 8 h led to a 2.7-log reduction in the number of CFU, while the addition of echinacoside (5.2 µg/mouse) caused an extra 1.4-log reduction in the number of CFU (Fig. 4c). This confirms that echinacoside can enhance the activity of tobramycin against established *P. aeruginosa* infections in murine lungs, in line with what was observed in vitro. Interestingly, the addition of echinacoside alone also resulted in a small (0.6-log) but significant decrease in the number of CFU compared to infected but untreated lungs (Fig. 4c). This direct effect of echinacoside was not observed in other models, and the underlying mechanism is unclear. Potentially this may be related to the antioxidant properties of echinacoside, which may improve immunity and host defenses^77,78^.

## Discussion

Different DGCs in *P. aeruginosa*, such as RoeA and SadC, make distinct contributions to surface-attached biofilm formation by controlling polysaccharide production and flagellar motility^79^. However, how different c-di-GMP-related enzymes contribute to non-surface attached aggregates in different clinically relevant environments is yet to be investigated in detail. Mutations in both *wspF* and *yfi* clusters are responsible for elevated intracellular c-di-GMP levels and the emergence of frequently isolated *P. aeruginosa* small-colony variants (SCVs) from CF lungs, which exhibit auto-aggregative behaviours^53,69,80–86^. In the present study, we showed that WspR, the structure of which has been widely used as the template for searching for novel DGC inhibitors based on virtual screening approaches, does not play a role in determining aggregate size in SCFM2 and, as a consequence, may not be a suitable target to reduce aggregation in vivo.

We subsequently quantified the expression of 40 genes in the c-di-GMP network in aggregated and planktonic *P. aeruginosa* cells, and this in a diverse collection of 32 strains. Specifically, aggregates were grown under microaerophilic conditions, providing oxygen levels similar to those experienced by *P. aeruginosa* in vivo and contributing to increased antibiotic tolerance^87^. However, our large-scale RT-qPCR analysis did not identify DGC or PDE encoding genes with an expression pattern much different from others, and only 28% of tested strains showed a decreased c-di-GMP level in planktonic cells compared to aggregates. It was previously reported that the c-di-GMP levels in mature *P. aeruginosa* biofilms are at least 2–3-fold higher than in planktonic cultures^58,59,88–90^ and the difference between these data and data obtained in the present study may be related to use of different media (LB or minimal medium in other studies), different culturing time and sampling techniques, as well as different metabolic rates in oxygen-limited environments. We subsequently tested whether the loss of *PA5442* and *siaD*, two genes encoding DGCs^65,91^, can reduce aggregation. Results indicated that SiaD does play an important role in auto-aggregation in SCFM2 which corresponds to its contribution to auto-aggregation in other environments^34,92^. *siaD* has not been identified as a frequently mutated gene in previous studies analysing multiple clinical isolates from CF patients^93,94^, unlike activating mutations in the *wsp* and *yfi* gene clusters^86^, and the amino acid sequence is 100% conserved in the 32 isolates investigated in this study (accession numbers for all strains are listed in Supplementary Table 2). The exact reasons for this are unclear but are likely a combination of 1) the relative difficulty to evolve a *siaD* gain-of-function mutant compared to a *wspR* gain-of-function; and 2) a possible physiological role for SiaA/B/C/D in *P. aeruginosa* lung survival that reduces the relative fitness of mutations in SiaD. These suggest that SiaD can serve as a drug target for designing new therapies against *P. aeruginosa* CF isolates. The fact that SiaD but not WspR or PA5442 is essential suggested that auto-aggregative behaviours of *P. aeruginosa* in physiologically relevant conditions may require the coordination of very specific c-di-GMP-related enzymes and signalling pathways. This postulation is supported by a recent report, where evolution-acquired mutations for *P. aeruginosa* occurred in two PDEs (BifA^95^ and DipA^90^) during passages in physiologically relevant media, but only mutations in *bifA* resulted in increased aggregation^96^. Interestingly, in the same study, mutations in the *siaA* promoter region emerged after PAO1 evolved in CF lung media, which led to its own overexpression^96^. SiaA/B/C/D constitute an operon. While the kinase SiaB maintains the phosphorylated state of SiaC, SiaA phosphatase induced by external stimuli can dephosphorylate SiaC. When SiaC remains unphosphorylated, it leads to SiaD activation and c‐di‐GMP production, thus promoting the formation of aggregates^35^. Mutation in *siaB* was previously reported to be related to SCV formation^82^. We tested the expression level of *siaA* using β-galactosidase assay, and the results showed that the increase in the expression level of *siaA* in aggregates vs*.* planktonic cells was comparable to the RT-qPCR results for *siaD* (Supplementary Fig. 1g; Supplementary RT-qPCR dataset). Therefore, while we proved in this study that SiaD is essential for *P. aeruginosa* aggregation in the CF environment during initial survival, its activity may be significantly increased due to mutations in *PsiaA* or *siaB* during evolution in chronic CF airways, suggesting the critical role of SiaA/B/C/D pathway in chronic infection establishment. Using molecular docking, we subsequently identified bimosiamose and echinacoside as potential inhibitors of SiaD and demonstrated that echinacoside successfully reduced the c-di-GMP level in PAO1 WT but not in the *ΔsiaD* mutant. Although we currently do not have data confirming that echinacoside directly interferes with the catalytic activity of SiaD exclusively and cannot rule out that echinacoside inhibits multiple DGCs or proteins containing both GGDEF and EAL domains given the structural similarities of active pockets among different GGDEF domains, our results suggest that echinacoside regulates global c-di-GMP levels *via* SiaD.

Echinacoside was recently shown to inhibit biofilm formation and potentiate vancomycin activity against methicillin-resistant *Staphylococcus aureus* by binding to a transpeptidase^97^. Here, we first demonstrated that a synergistic killing effect of echinacoside and tobramycin can be observed for aggregates formed by >80% tested *P. aeruginosa* CF isolates (including resistant and mucoid strains) in mucus-like environments, with aggregate sizes similar to most of those observed in CF patients (5-100 μm in diameter)^24^. Notably, this potentiating effect was not observed for the *ΔsiaD* mutant in SCFM2, which is consistent with measured c-di-GMP levels and provides additional evidence that echinacoside affects SiaD activity. Moreover, echinacoside also potentiated the efficacy of tobramycin against *P. aeruginosa* biofilms on the surface of 3-D A549 cells and in agar beads in murine lungs, suggesting echinacoside (and other compounds with similar mode of action) has potential as an adjunctive therapy to treat *P. aeruginosa* embedded in the CF airway mucus layer. Intriguingly, for aggregates grown in the SCFM2 and 3-D A549 cell models, this potentiating effect was concentration-dependent and disappeared when high concentrations of echinacoside were used, consistent with its influence on aggregate sizes.

In conclusion, this study, for the first time, systematically investigated the expression profiles of c-di-GMP-related genes among various *P. aeruginosa* CF isolates in a pre-clinical model, identified SiaD as a druggable target, and discovered the antibiotic-potentiating activity of echinacoside against *P. aeruginosa* aggregates formed in different in vitro and in vivo models. Our findings indicate that therapies based on a combination of a compound that can reduce intracellular c-di-GMP levels with an antibiotic have the potential to show anti-biofilm activity in vivo. Finally, our data also reinforce the importance of using clinically relevant biofilm and infection models for future drug discovery as stated by many researchers in recent years^3,27,98^.

## Supplementary information

Supplementary files

PA0169 - MCE Top 200 Compound Details

qPCR raw data – updated

## Data Availability

The genome sequences of all CF isolates from Belgium are deposited in the NCBI GenBank with accession number PRJNA1072279. Source data, including the existence of c-di-GMP-related genes in each strain, the MIC (µg/mL) of tobramycin and meropenem against different strains, RT-qPCR data, aggregate sizes in each micrograph, and virtual docking scores of 200 compounds against the SiaD active site, are provided in this paper in supplemental data.
