# Supplementary material for: Echinacoside reduces intracellular c-di-GMP levels and potentiates tobramycin activity against Pseudomonas aeruginosa biofilm aggregates
Source: NPJ Biofilms Microbiomes. 2025 Mar 7;11:40. doi: 10.1038/s41522-025-00673-2 (PMC11889090; doi:10.1038/s41522-025-00673-2)
Supplement: Supplementary file 1 — Supplementary files [file 41522_2025_673_MOESM1_ESM.pdf]

Supplementary Figure 1

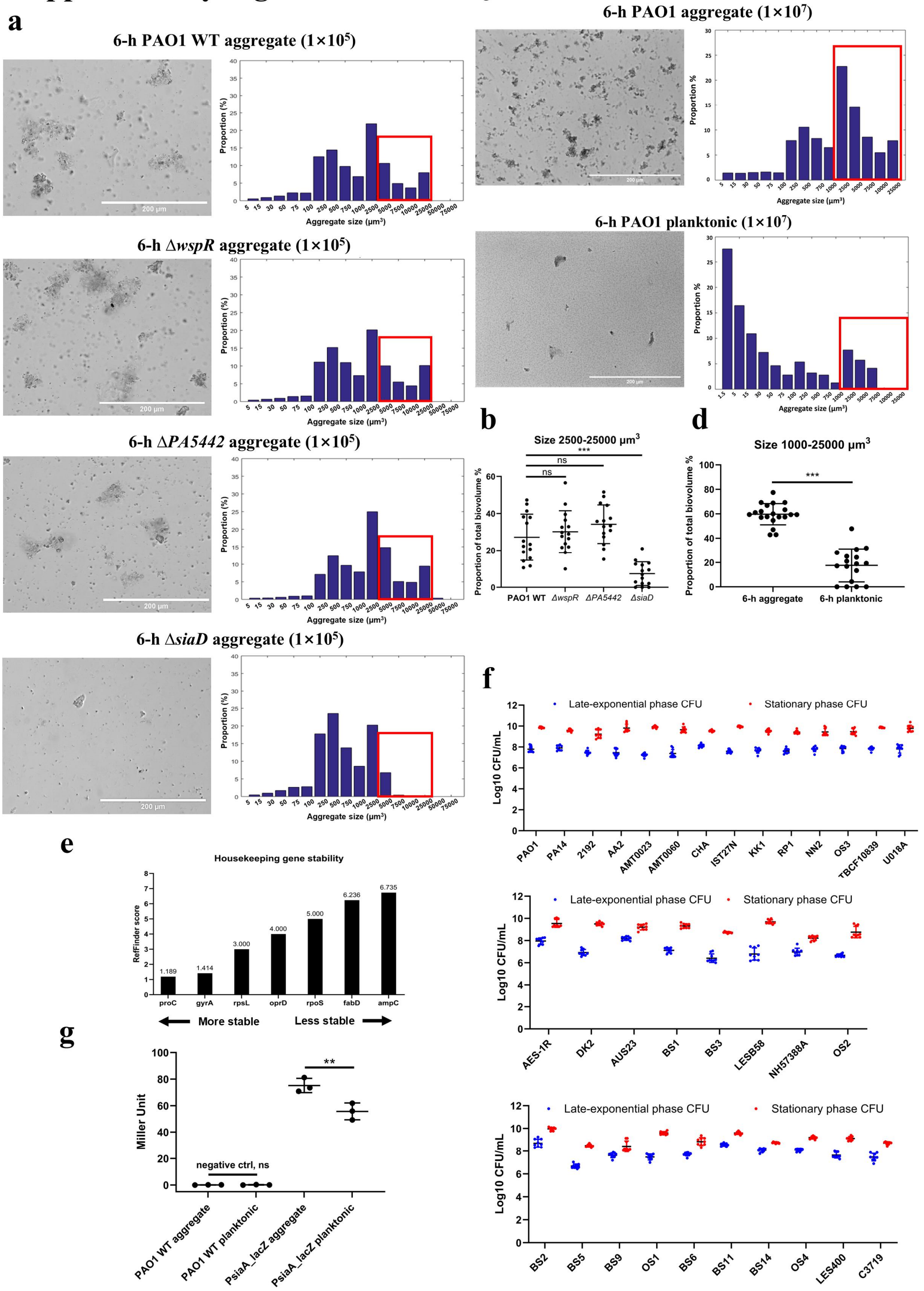

Supplementary Figure 1. Cell morphologies, cell densities, and the stability of different housekeeping genes in *P. aeruginosa* aggregates and/or planktonic cultures. a) Representative micrographs of *P. aeruginosa* PAO1 WT, *ΔwspR*, *ΔPA5442,* and *ΔsiaD* aggregates (initial inoculum 1×10^5 CFU/mL) grown in SCFM2 for 6 hrs (Scale bar = 200 μm) and the corresponding distribution of the sizes of aggregates. The

biovolume (μm^3) of aggregates were grouped into 16 categories. Histograms represent the mean proportion value of all samples collected from 3 individual experiments (n=3), where micrographs were obtained from at least 3 random locations in each sample. Numerical data of mean proportions and standard deviation are shown in Supplementary Table 1. **b)** Proportion of aggregates in a) with sizes ranging from 2500 to 25000 μm^3 in total biovolume. ***, p<0.001 (Student's t-test). **c)** Representative micrographs of *P. aeruginosa* PAO1 WT aggregates (in SCFM2) and planktonic cells (in SCFM2 without mucin and DNA) grown for 6 hrs with an initial inoculum size of 1×10^7 CFU/mL (Scale bar = 200 μm) and distribution of the size of 6-h PAO1 WT aggregates and planktonic cells. Histograms represent the mean proportion value of all samples collected from 3 individual experiments (n=3), where micrographs were obtained from at least 3 random locations in each sample. Numerical data and standard deviations are shown in Supplementary Table 1. **d)** Proportion of aggregates in c) with sizes ranging from 1000 to 25000 μm^3 in total biovolume. ***, p<0.001 (Student's t-test). **e)** Stability of expression of 7 candidate reference genes for qPCR normalization as determined with RefFinder. The stability index of each gene is shown above each bar. **f)** Cell density of late-exponential phase aggregate culture of each strain after the desired incubation time in SCFM2. The starting inoculum concentrations for all strains were 1×10^5 CFU/mL, and the CFU counts were determined by agar plating. The late-exponential phase growth was defined when the CFU count was 1-2-log lower than the corresponding stationary phase cultures. **g)** The relative expression level of *siaA* in aggregates *vs* planktonic cells determined by β-galactosidase assay and quantified as Miller Units. PAO1 WT without the *lacZ* reporter plasmid was used as the negative control.

Supplementary Figure 2

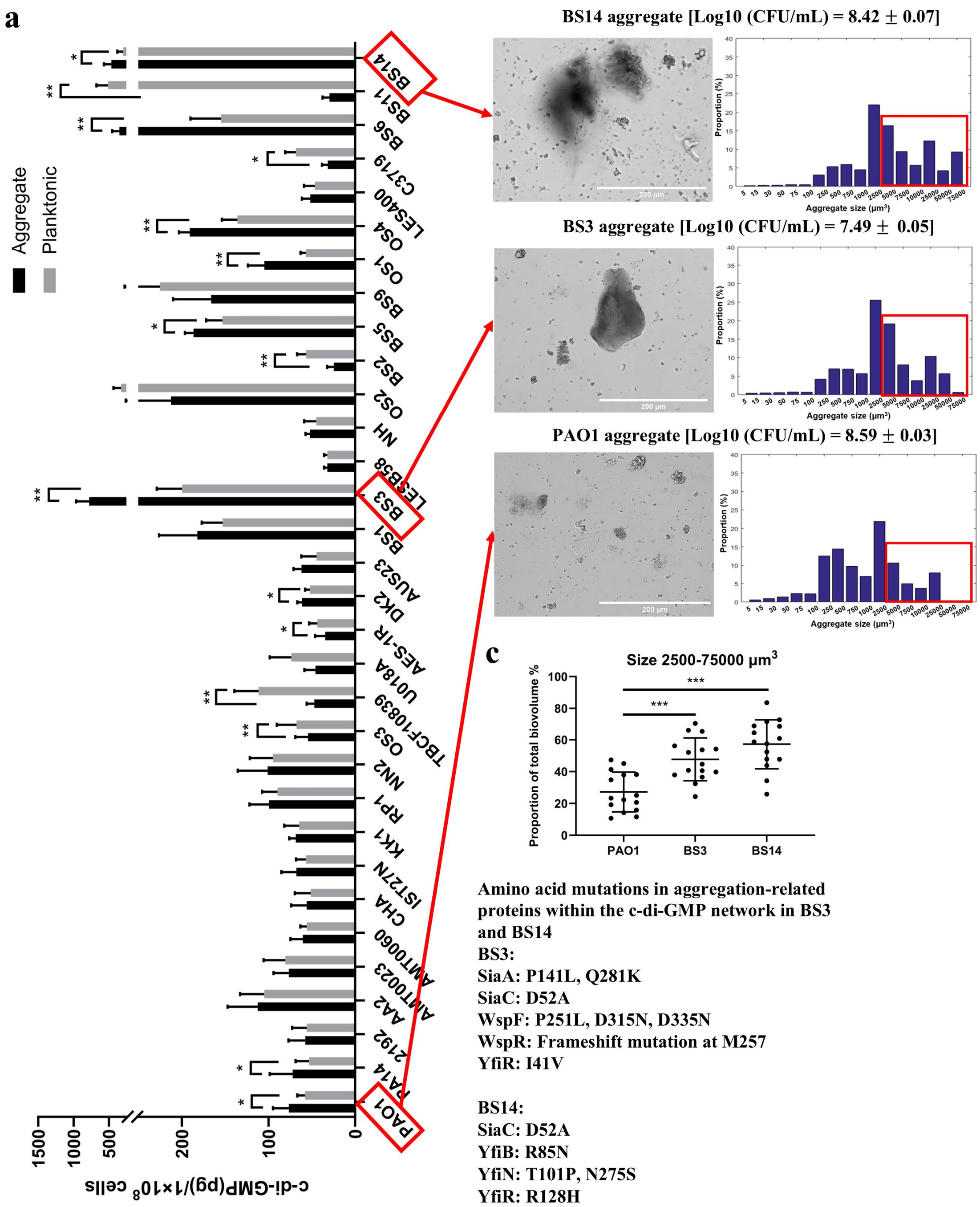

Supplementary Figure 2. Intracellular c-di-GMP levels and aggregation patterns of *P. aeruginosa* clinical strains. a) Intracellular c-di-GMP levels for 32 *P. aeruginosa* strains grown as aggregates (in SCFM2) or planktonic cells (in SCFM2 without DNA and mucin). Intracellular c-di-GMP concentrations (as determined by ELISA) were normalized to CFU counts (determined by plating). Data were acquired from 3 independent experiments with 2 technical replicates and the mean values were shown. Error bars indicate standard deviation. *, $p<0.05$; **, $p<0.01$ (Ratio paired t-test

between two groups). **b)** Representative micrographs of *P. aeruginosa* PAO1, BS3, and BS14 aggregates (initial inoculum 1×10^5 CFU/mL) grown in SCFM2 until the late-exponential phase (Scale bar = 200 µm) and the corresponding distribution of the size of aggregates (final cell density at the point of sampling labelled above, n=3). The biovolume (μm^3) of aggregates were grouped into 16 categories. Histograms represent the mean proportion value of all samples collected from 3 individual experiments (n=3), where micrographs were obtained from at least 3 random locations in each sample. Numerical data of mean proportions and standard deviation are shown in Supplementary Table 1. **c)** Proportion of aggregates in b) with sizes ranging from 2500 to 75000 μm^3 in total biovolume. ***, $p<0.001$ (Student's t-test).

Supplementary Figure 3

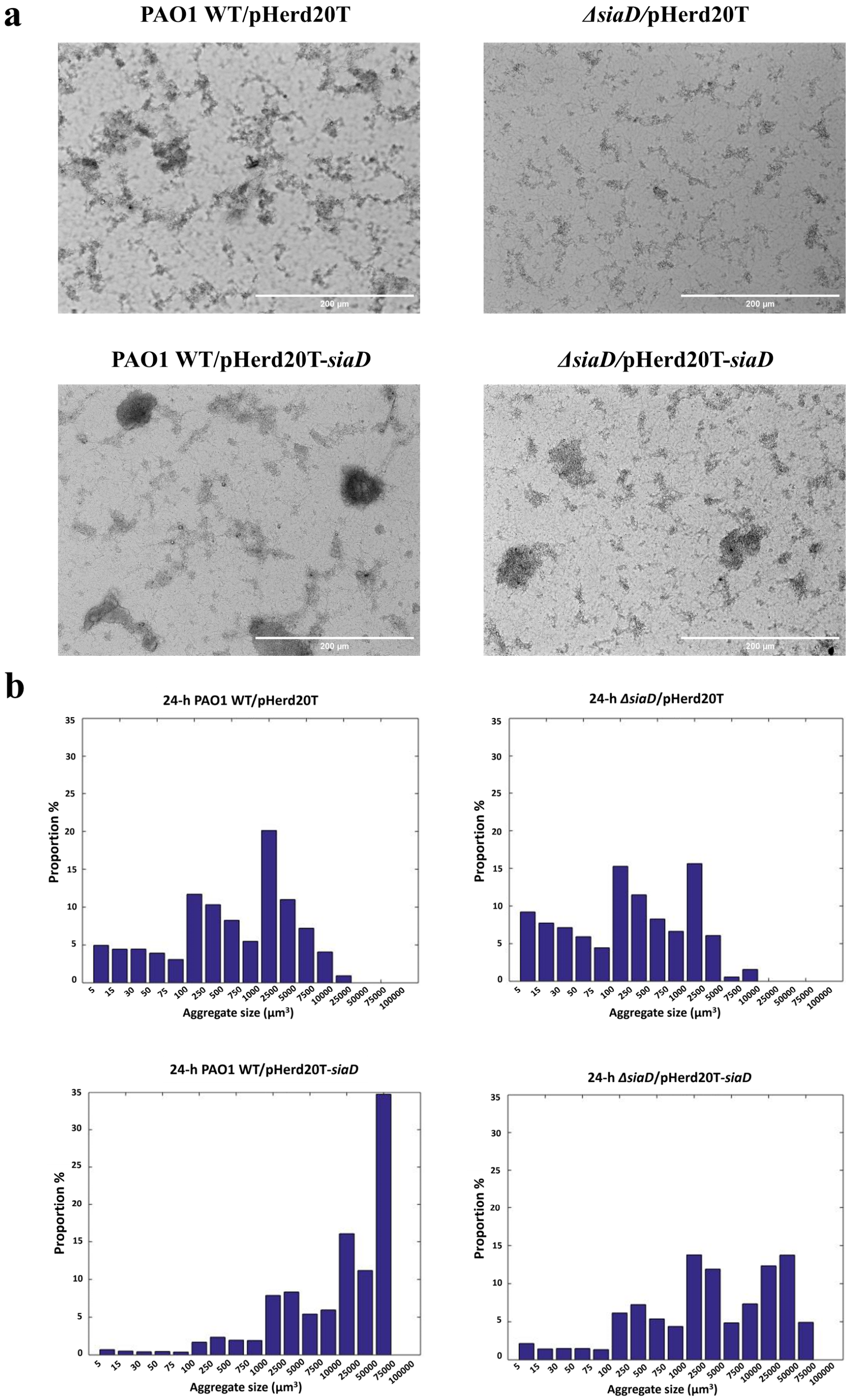

Supplementary Figure 3. The aggregate morphologies of PAO1 WT and *ΔsiaD* complemented with empty vector or *siaD*. a) Representative micrographs of PAO1 WT/pHerd20T, *ΔsiaD*/pHerd20T, PAO1 WT/pHerd20T-*siaD* and *ΔsiaD*/pHerd20T-*siaD* aggregates grown for 24 h in SCFM2. Scale bar = 200 µm. Plasmids were

maintained by adding 200 μg/mL carbenicillin and expression was induced by 0.5% (v/v) arabinose. **b)** Distribution of the size of 24-h PAO1 WT/pHerd20T, *ΔsiaD*/pHerd20T, PAO1 WT/pHerd20T-*siaD* and *ΔsiaD*/pHerd20T-*siaD* aggregates grown in SCFM2. The biovolume (μm^3) of single cells and aggregates were grouped into 17 categories. Histograms represent the mean proportion value of all samples collected from 3 individual experiments (n=3), where micrographs were obtained from at least 3 random locations in each sample. Numerical data of mean proportions and standard deviation are shown in Supplementary Table 1.

Supplementary Figure 4

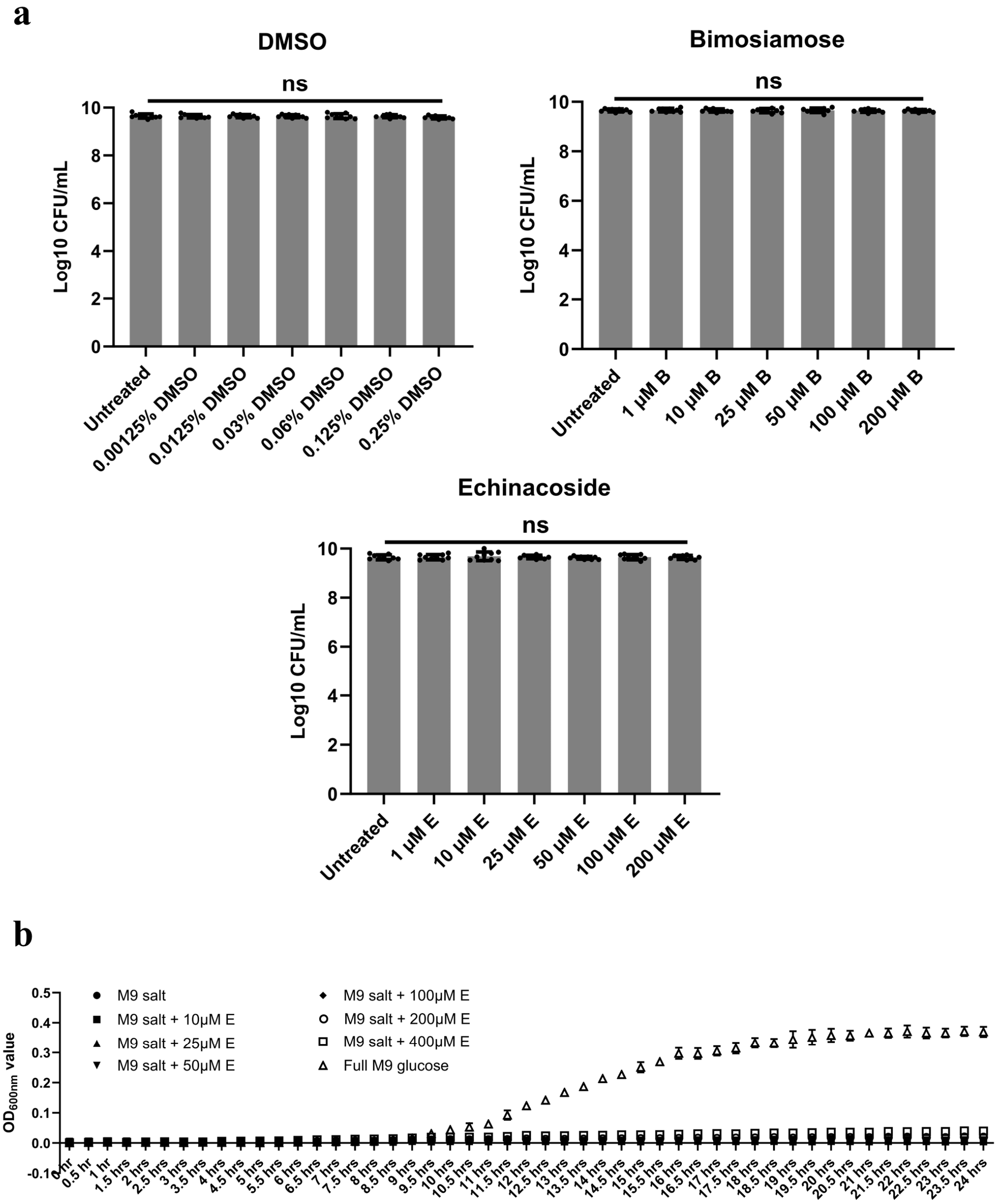

Supplementary Figure 4. Potential anti-biofilm compounds do not interfere with the growth of PAO1. a) Bactericidal tests for DMSO (solvent for bimosiamose), bimosiamose, and echinacoside against 6-h pre-established PAO1 aggregates grown in SCFM2. Data are expressed as the mean number of CFU remaining after an additional 18-h incubation (3 independent experiments with 3 technical replicates. No statistically significant difference was found comparing untreated and treated samples (One-way

ANOVA). **b)** Echinacoside is not a nutrient source that promotes *P. aeruginosa* cell growth. 1×10^5 CFU/mL PAO1 WT cells were inoculated into M9 minimal medium without glucose, M9 minimal medium without glucose but with 25, 50, 100, 200, and 400 µM echinacoside (the same concentration range used in combination treatment assay), and M9 minimal medium (with glucose). The OD_{600nm} measurement was performed every 30 mins over 24-h incubation at 37°C, and the growth curve was plotted with data from 3 independent experiments with 3 technical replicates (n=3).

Supplementary Figure 5

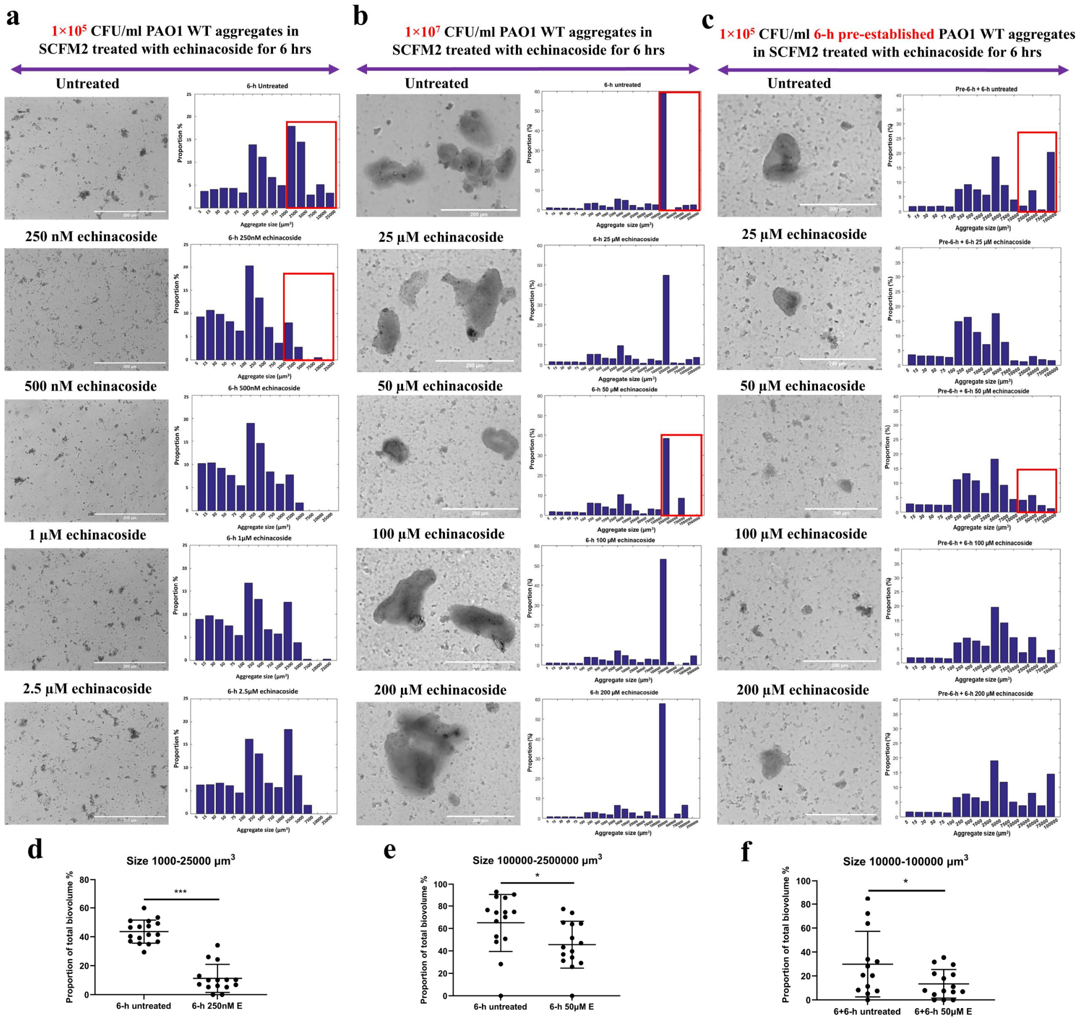

Supplementary Figure 5. Echinacoside inhibits aggregation in a concentration-dependent manner. a) Representative micrographs of 6-h *P. aeruginosa* PAO1 aggregates (initial inoculum 1×10^5 CFU/mL) grown in SCFM2 treated with different concentrations of echinacoside (Scale bar = 200 µm) and the corresponding distribution of the size of aggregates. The biovolume (µm^3) of aggregates were grouped into 14

categories. **b)** Representative micrographs of 6-h *P. aeruginosa* PAO1 aggregates (initial inoculum 1×10^7 CFU/mL) grown in SCFM2 treated with different concentrations of echinacoside (Scale bar = 200 μm) and the corresponding distribution of the size of aggregates. The biovolume (μm^3) of aggregates were grouped into 20 categories. **c)** Representative micrographs of 6-h pre-established *P. aeruginosa* PAO1 aggregates (initial inoculum 1×10^5 CFU/mL) grown in SCFM2 treated with different concentrations of echinacoside for another 6 hrs (Scale bar = 200 μm) and the corresponding distribution of the size of aggregates. The biovolume (μm^3) of aggregates were grouped into 16 categories. Histograms represent the mean proportion value of all samples collected from 3 individual experiments (n=3), where micrographs were obtained from at least 3 random locations in each sample. Numerical data of mean proportions and standard deviation are shown in Supplementary Table 1. **d)** Proportion of aggregates in (a) with sizes ranging from 1000 to 25000 μm^3 in total biovolume. ***, $p<0.001$ (Student's t-test). **e)** Proportion of aggregates in b) with sizes ranging from 100000 to 2500000 μm^3 in total biovolume. *, $p<0.05$. **f)** Proportion of aggregates in c) with sizes ranging from 10000 to 100000 μm^3 in total biovolume. *, $p<0.05$

Supplementary Figure 6

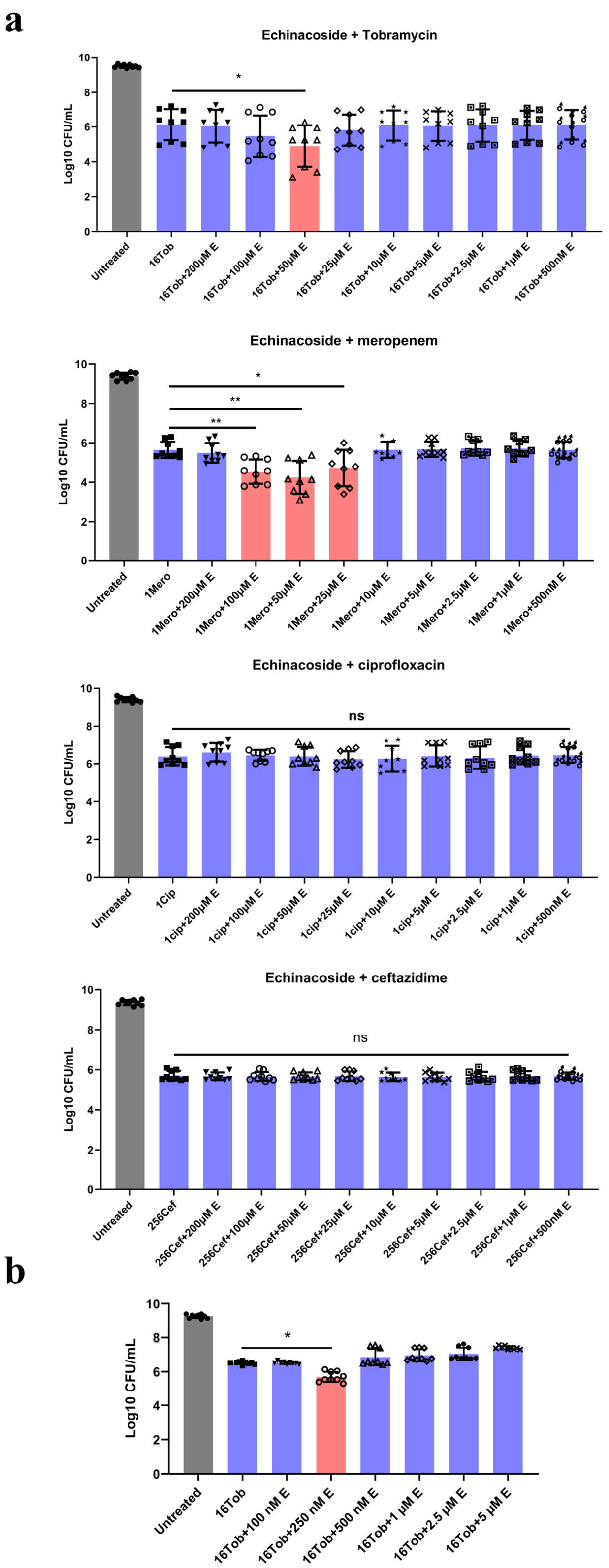

Supplementary Figure 6. Combination treatments of echinacoside and different antibiotics against *P. aeruginosa* PAO1 aggregates. a) Antimicrobial activity of the

combination of echinacoside with tobramycin, meropenem, ciprofloxacin, and ceftazidime, respectively, against 6-h pre-established *P. aeruginosa* PAO1 aggregates grown in SCFM2. Data are expressed as the mean number of CFU remaining after an additional 18-h incubation (3 independent experiments with 3 technical replicates; error bars indicate standard deviation). Red bars highlighted the successful combination treatment that potentiated the efficacy of corresponding antibiotics. *, $p<0.05$; **, $p<0.01$ (Student's t-test). **b)** Antimicrobial activity of tobramycin against aggregates pre-treated with echinacoside for 6 hours. Data are expressed as the mean number of CFU remaining after an additional 18-h incubation with tobramycin treatment (3 independent experiments with 3 technical replicates; error bars indicate standard deviation). Red bars highlighted the successful combination treatment that potentiated the efficacy of corresponding antibiotics. *, $p<0.05$ (Student's t-test).

Supplementary Figure 7

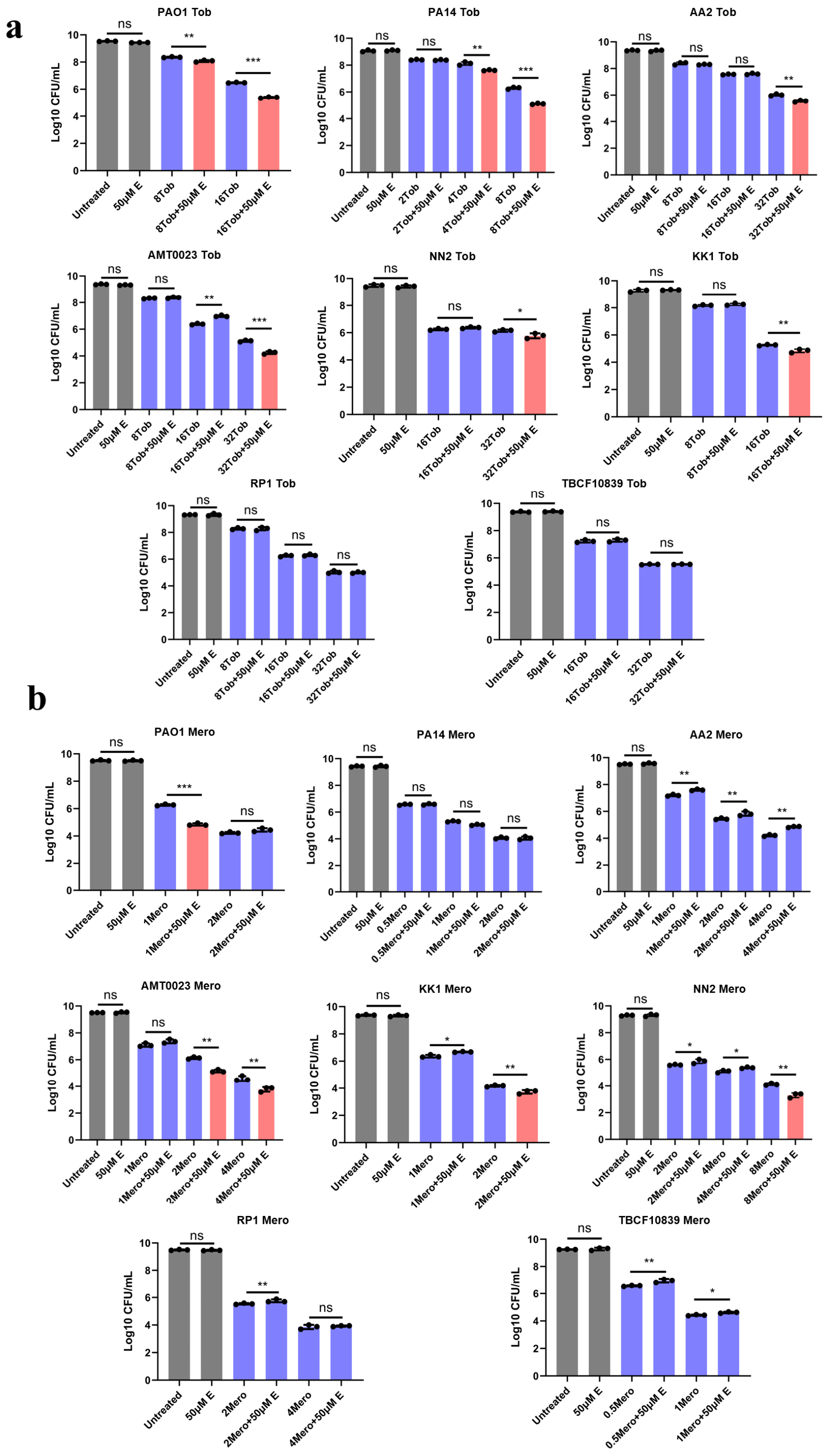

Supplementary Figure 7. Combination treatments of echinacoside and tobramycin/meropenem against different *P. aeruginosa* strains. Efficacy test for the

combination treatment of 50 μM echinacoside with different concentrations of **a)** tobramycin or **b)** meropenem against pre-established aggregates formed by 8 different *P. aeruginosa* strains in SCFM2. Data are expressed as the mean number of CFU remaining after an additional 18-h incubation (3 independent experiments with 3 technical replicates; error bars indicate standard deviation). Red bars highlighted the successful combination treatment that potentiated the efficacy of corresponding antibiotics. *, $p<0.05$; **, $p<0.01$; ***, $p<0.001$ (Student's t-test).

Supplementary Figure 8

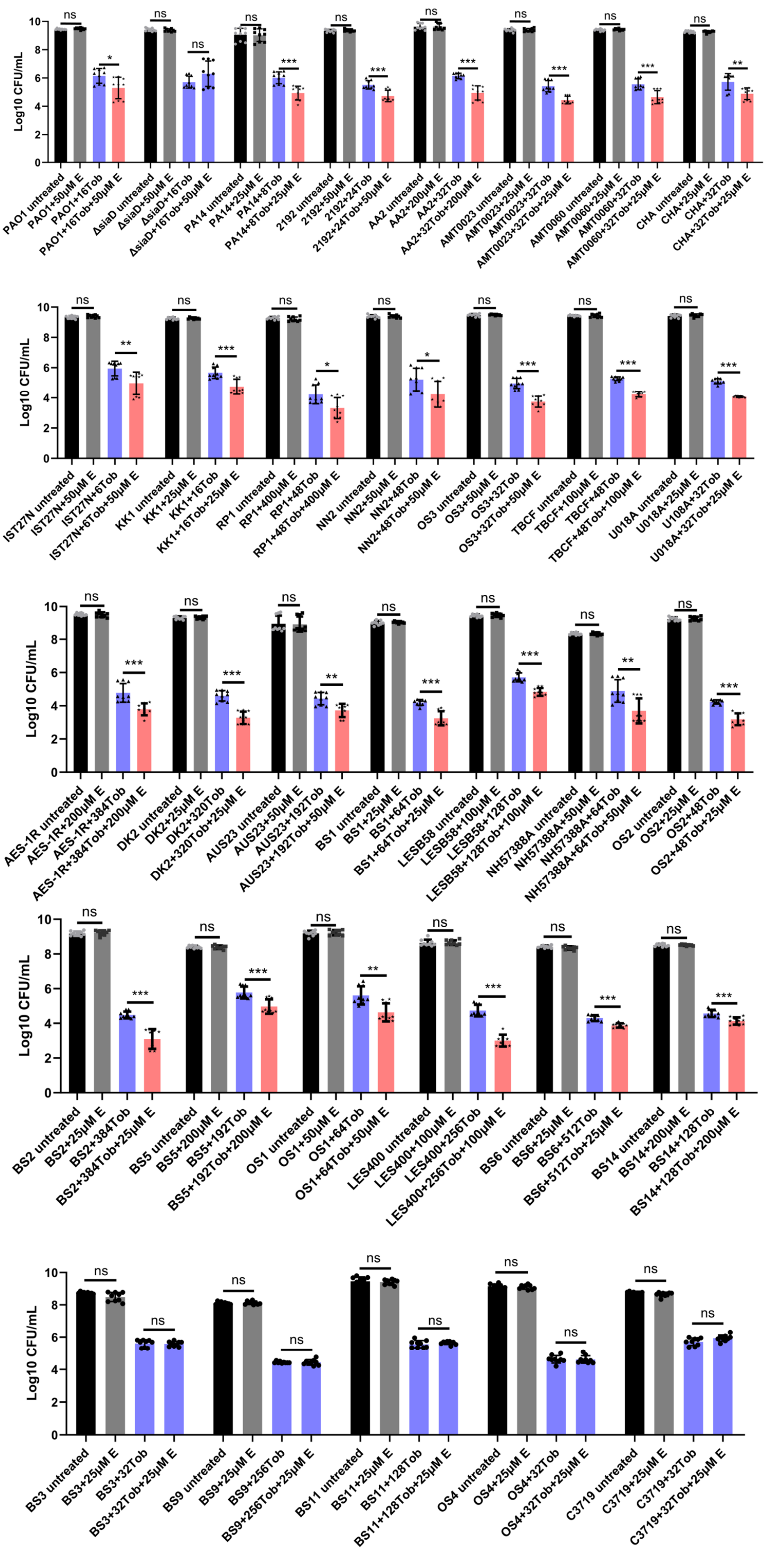

Supplementary Figure 8. The synergistic killing effect of echinacoside and tobramycin against aggregates formed by different *P. aeruginosa* strains in SCFM2. The optimal dosages for successful combination treatments where echinacoside potentiated the efficacy of tobramycin against pre-established aggregates grown in SCFM2 for 27 *P. aeruginosa* strains and the representative data for unsuccessful combination treatments where echinacoside cannot potentiate the efficacy of tobramycin at any tested concentrations. Data are expressed as the mean number of CFU remaining after an additional 18-h incubation (3 independent experiments with 3 technical replicates; error bars indicate standard deviation). *, $p<0.05$; **, $p<0.01$; ***, $p<0.001$; ns, not significant (Student's t-test).

Supplementary Figure 9

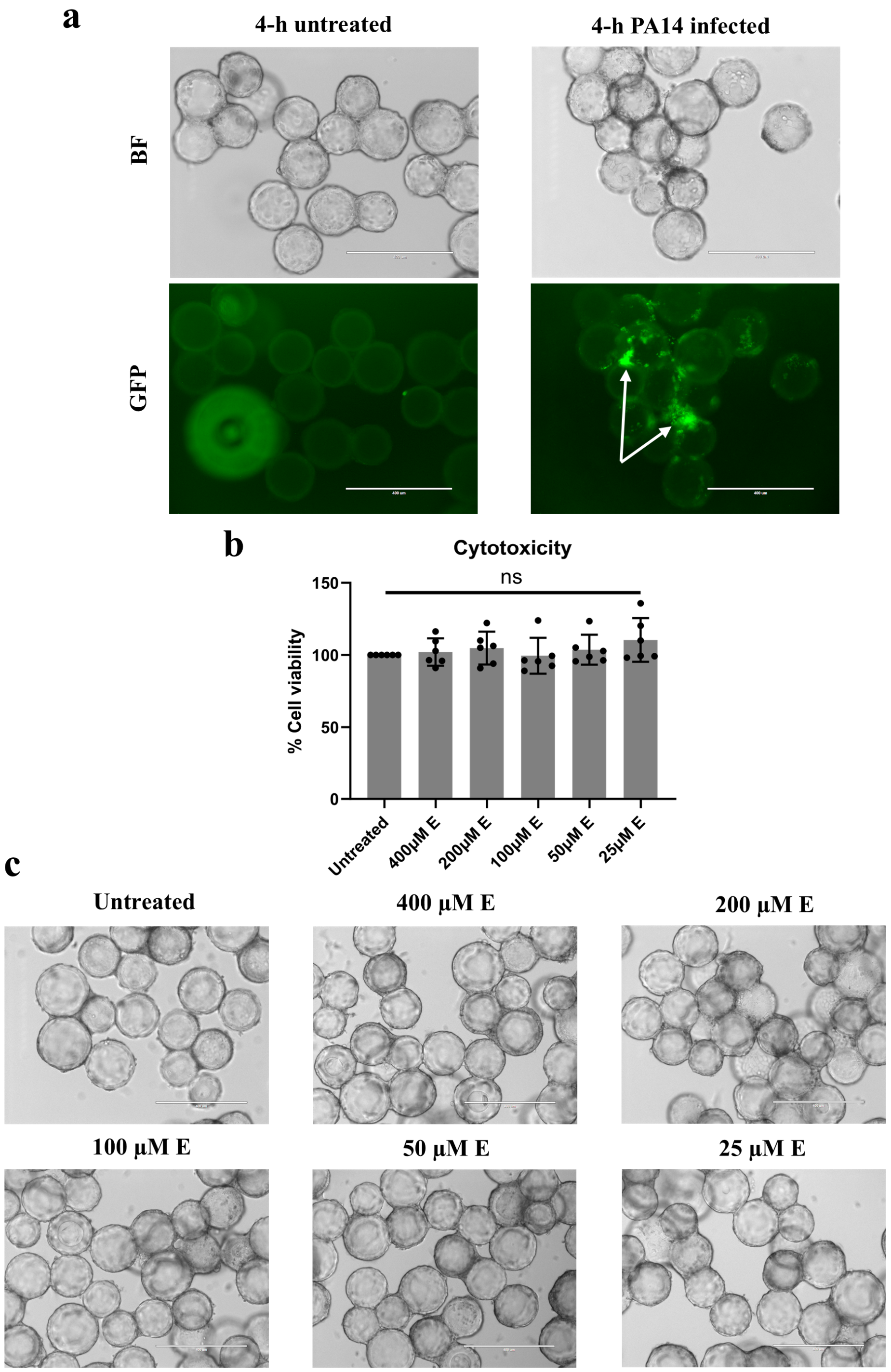

Supplementary Figure 9. Echinacoside is not cytotoxic to human A549 cells. a) Representative transmission light micrographs (upper panel) of 3-D A549 cells attached to/detached from collagen-treated carrier beads and fluorescent micrographs (lower panel) of GFP-tagged PA14 attached to A549 cells. After 4-h infection, PA14 aggregates (highlighted with white arrows) were found attached to A549 cells with

strong fluorescent signals. Healthy cells in the untreated group showed a smooth morphology and remained firmly attached to beads, while cells infected with PA14 were stressed (rough and patchy morphology at the surface of beads). Scale bar = 400 μm. **b)** Cytotoxicity test for different concentrations of echinacoside. The viability of 3-D A549 cells challenged with different concentrations of echinacoside for 18 hrs was measured by LDH assay and normalized to untreated groups. Data were acquired from 6 independent experiments with 4 technical replicates. No statistically significant difference was found comparing untreated and treated cells (One-way ANOVA). **c)** Representative transmission light micrographs of 3-D A549 cells attached to collagen-treated carrier beads challenged with different concentrations of echinacoside. Healthy cells in all groups showed a smooth morphology and remained firmly attached to beads without difference in attachment patterns. Scale bar = 400 μm.

Supplementary Table 1. Mean fraction (%) of aggregates within each size category in total biovolume. SD, standard deviation. N=3

6-h aggregates vs planktonic (initial inoculum 1×10^{7} CFU/mL)				
Size (μm^3)	aggregate		planktonic	
	mean	SD	mean	SD
1.5-5	N/A	N/A	27.64	10.31
5-15	1.44	0.48	16.44	4
15-30	1.39	0.43	10.92	3.94
30-50	1.52	0.48	7.26	2.73
50-75	1.64	0.49	4.65	2.25
75-100	1.48	0.5	2.82	1.56
100-250	7.89	3.04	5.38	3.21
250-500	10.56	2.59	3.23	1.55
500-750	8.31	2.36	2.81	2.14
750-1000	6.5	2.1	1.27	1.66
1000-2500	22.77	4.95	7.72	7.73
2500-5000	14.56	3.99	5.74	7.18
5000-7500	8.58	5.3	4.14	6.99
7500-10000	5.48	4.29	0	0
10000-25000	7.87	5.59	0	0
25000-50000	0	0	0	0

6-h aggregates (initial inoculum 1×10^{5} CFU/mL)								
Size (μm^3)	WT		*ΔwspR*		*ΔPA5442*		*ΔsiaD*	
	mean	SD	mean	SD	mean	SD	mean	SD
5-15	0.55	0.42	0.43	0.21	0.36	0.17	0.49	0.33
15-30	0.92	0.84	0.67	0.37	0.42	0.17	0.98	0.87
30-50	1.34	1.23	0.93	0.58	0.62	0.35	1.72	1.28
50-75	2.25	2.19	1.43	0.78	0.91	0.54	2.63	1.81
75-100	2.20	2.11	1.59	0.83	1.02	0.59	2.81	1.83
100-250	12.53	9.41	11.11	5.64	7.14	4.10	17.76	8.44
250-500	14.44	7.17	15.20	4.75	12.41	5.36	23.54	4.96
500-750	9.77	4.06	10.98	2.50	9.69	3.05	13.81	2.71
750-1000	6.88	2.09	7.32	2.69	7.84	1.94	8.59	2.98
1000-2500	21.89	11.44	20.17	5.44	24.97	5.64	20.25	8.09
2500-5000	10.65	7.30	10.06	5.42	14.82	5.09	6.78	5.71
5000-7500	4.91	4.75	5.51	4.21	5.09	5.08	0.44	0.94
7500-10000	3.67	3.92	4.45	4.39	4.87	3.75	0.20	0.77
10000-25000	7.99	5.61	10.15	5.47	9.47	5.81	0	0
25000-50000	0.00	0	0	0	0.37	1.44	0	0
50000-75000	0.00	0	0	0	0	0	0	0

6-h aggregates (initial inoculum 1×10^5 CFU/mL)										
Size (μm^3)	Untreated		250 nM E		500 nM E		1 μM E		2.5 μM E	
	mean	SD	mean	SD	mean	SD	mean	SD	mean	SD
5-15	3.67	1.2	9.29	2.93	10.21	4.5	9.21	4.31	6.24	2.28
15-30	4.07	1.78	10.69	2.6	10.34	2.68	9.87	3.9	6.31	1.83
30-50	4.37	1.79	9.85	1.82	9.18	2.61	8.81	3.16	6.64	2.15
50-75	4.32	1.34	8.26	1.27	7.65	1.88	7.59	2.38	6.11	1.88
75-100	3.32	0.76	6.25	0.84	5.44	1.83	5.5	1.74	4.53	2.22
100-250	13.84	3.29	20.37	3.11	18.99	3.58	16.69	3.95	16.26	3.6
250-500	11.15	3.51	13.36	2.82	14.56	4.06	12.83	2.6	12.99	3.37
500-750	6.72	2.45	7.03	1.76	8.41	4.41	6.82	2.97	6.65	1.73
750-1000	4.93	1.85	3.63	2.11	5.77	4.12	5.62	2.73	5.71	3.28
1000-2500	17.89	5.23	8	6.1	7.76	5.11	12.12	9	18.38	9.19
2500-5000	14.44	9.06	2.77	3.84	1.71	3.03	4.2	5.29	8.29	7.39
5000-7500	2.87	3.86	0	0	0	0	0.44	1.09	1.88	3.85
7500-10000	5.13	5.09	0.51	1.97	0	0	0	0	0	0
10000-25000	3.28	4.37	0	0	0	0	0.3	1.33	0	0

6-h aggregates (initial inoculum 1×10^7 CFU/mL)										
Size (μm^3)	Untreated		25 μM E		50 μM E		100 μM E		200 μM E	
	mean	SD	mean	SD	mean	SD	mean	SD	mean	SD
5-15	1.16	0.95	1.54	0.71	1.84	0.58	1.07	0.61	0.79	0.51
15-30	0.97	0.88	1.50	0.69	1.72	0.56	1.05	0.60	0.79	0.56
30-50	0.97	0.94	1.45	0.66	1.68	0.56	1.03	0.57	0.77	0.53
50-75	0.89	0.90	1.47	0.68	1.71	0.58	1.01	0.56	0.76	0.50
75-100	0.76	0.75	1.28	0.59	1.33	0.40	0.91	0.56	0.63	0.41
100-250	3.25	4.11	5.26	2.40	6.14	2.25	3.98	2.35	2.86	1.97
250-500	3.47	5.10	5.28	2.66	5.94	2.87	3.87	2.62	3.04	2.32
500-750	2.16	2.62	3.38	1.98	4.23	2.37	2.71	1.77	2.28	1.81
750-1000	1.41	1.36	3.04	2.16	3.29	1.93	2.10	1.16	1.78	1.36
1000-2500	5.54	4.84	9.58	6.06	10.35	5.45	7.17	5.02	6.48	5.60
2500-5000	4.90	3.44	4.61	2.68	5.65	3.51	4.83	3.92	4.67	3.09
5000-10000	2.34	1.49	2.84	1.96	2.33	1.96	2.78	2.65	2.80	1.54
10000-25000	1.27	1.60	0.84	1.03	0.82	1.16	1.37	1.84	1.13	1.25
25000-50000	3.15	2.75	2.84	2.87	3.20	2.94	3.03	4.49	3.34	2.74
50000-75000	2.62	3.94	2.09	4.57	2.84	4.61	2.79	6.25	1.36	2.04
75000-100000	58.93	29.35	44.69	27.37	38.38	26.99	53.22	28.82	57.77	31.82
100000-250000	0.00	0.00	1.04	4.03	0.00	0.00	1.40	5.42	0.00	0.00
250000-500000	1.26	4.88	7.26	19.82	8.55	22.57	0.00	0.00	2.18	8.44
500000-750000	2.37	9.19	0.00	0.00	0.00	0.00	1.03	4.00	6.59	18.55
750000-1000000	2.58	10.00	0.00	0.00	0.00	0.00	4.67	18.10	0.00	0.00
1000000-2500000	0.00	0.00	0.00	0.00	0.00	0.00	0.00	0.00	0.00	0.00

Pre-6-h aggregate + 6-h treatments (initial inoculum 1×10^5 CFU/mL)										
Size (μm^3)	Untreated		25 μM E		50 μM E		100 μM E		200 μM E	
	mean	SD	mean	SD	mean	SD	mean	SD	mean	SD
5-15	1.72	0.75	3.58	0.71	2.88	0.75	1.90	0.51	1.69	0.72
15-30	1.79	0.79	3.21	0.66	2.59	0.64	1.83	0.53	1.61	0.68
30-50	1.72	0.76	3.19	0.68	2.57	0.71	1.83	0.54	1.60	0.71
50-75	1.83	0.81	3.02	0.64	2.45	0.92	1.72	0.43	1.58	0.64
75-100	1.60	0.71	2.74	0.74	2.43	1.03	1.55	0.51	1.38	0.57
100-250	7.59	3.34	14.84	4.02	11.18	4.35	7.10	2.21	6.58	2.44
250-500	9.22	4.12	16.29	3.57	13.26	5.02	8.77	2.63	7.80	3.42
500-750	7.43	3.77	11.20	2.20	10.78	3.79	7.74	2.28	6.53	2.53
750-1000	5.63	2.99	7.06	1.53	6.46	1.78	5.96	1.72	5.26	2.26
1000-2500	18.68	7.67	17.52	3.98	18.20	5.28	19.54	4.31	19.01	5.87
2500-5000	8.93	3.97	7.82	4.30	9.27	4.37	14.07	2.78	11.78	4.27
5000-7500	3.96	2.64	1.58	2.23	4.41	5.60	8.90	2.91	5.06	2.71
7500-10000	1.89	1.72	1.27	2.11	4.12	3.76	3.68	3.04	3.81	3.02
10000-25000	7.14	4.99	3.05	5.21	5.77	5.92	8.97	5.66	8.03	4.04
25000-50000	0.61	2.18	1.98	5.23	2.34	4.92	1.92	3.47	3.79	5.08
50000-75000	20.26	29.32	1.64	6.35	1.26	4.90	4.51	8.65	14.50	20.94
75000-100000	0.00	0.00	0.00	0.00	0.00	0.00	0.00	0.00	0.00	0.00

late-exponential phase aggregates (initial inoculum 1×10^5 CFU/mL)						
Size (μm^3)	WT		BS3		BS14	
	mean	SD	mean	SD	mean	SD
5-15	0.55	0.42	0.42	0.12	0.21	0.11
15-30	0.92	0.84	0.49	0.20	0.29	0.23
30-50	1.34	1.23	0.57	0.27	0.36	0.31
50-75	2.25	2.19	0.72	0.50	0.48	0.55
75-100	2.20	2.11	0.69	0.46	0.48	0.68
100-250	12.53	9.41	4.21	2.48	3.09	4.42
250-500	14.44	7.17	7.00	3.22	5.32	4.50
500-750	9.77	4.06	6.92	2.51	5.92	4.59
750-1000	6.88	2.09	5.71	1.81	4.50	2.29
1000-2500	21.89	11.44	25.55	5.86	22.01	7.07
2500-5000	10.65	7.30	19.16	5.58	16.39	6.28
5000-7500	4.91	4.75	8.09	3.73	9.39	4.20
7500-10000	3.67	3.92	3.81	3.36	5.72	3.05
10000-25000	7.99	5.61	10.37	4.27	12.27	7.73
25000-50000	0.00	0	5.68	5.15	4.24	4.35
50000-75000	0.00	0	0.64	1.72	9.32	6.25

24-h aggregates (initial inoculum 1×10^5 CFU/mL)																		
Size (μm^3)	PAO1 WT (1)		*ΔwspR*		PAO1 WT (2)		*Δpa5442*		*ΔsiaD*		WT/EV		WT/*siaD*		*ΔsiaD* /EV		*ΔsiaD/siaD*	
	mean	SD	mean	SD	mean	SD	mean	SD	mean	SD	mean	SD	mean	SD	mean	SD	mean	SD
5-15	1.49	1.04	1.65	0.99	1.95	1.22	1.76	1.09	16.78	9.6	4.95	4.01	0.71	0.22	9.2	5.05	2.14	1.17
15-30	1.37	1.23	1.51	0.86	1.86	1.08	1.63	0.82	12.19	4.32	4.45	3.07	0.52	0.19	7.74	2.7	1.43	0.57
30-50	1.3	1.09	1.49	0.98	1.78	0.99	1.6	0.67	11.28	4.04	4.46	2.85	0.42	0.15	7.13	2.19	1.5	0.62
50-75	1.36	0.95	1.36	0.86	1.73	1.03	1.75	0.84	9.64	3.57	3.93	2.03	0.46	0.24	5.92	1.66	1.49	0.54
75-100	1.17	0.8	1.33	0.68	1.47	0.85	1.44	0.74	6.12	2.67	3.08	1.29	0.39	0.19	4.45	1.17	1.34	0.5
100-250	5.66	3.62	5.53	2.18	6.17	2.99	6.95	3.39	23.29	7.9	11.71	3.51	1.71	0.76	15.28	2.66	6.15	2.29
250-500	6.77	4.12	6.37	1.95	6.21	2.57	7.97	3.71	11.51	8.35	10.32	2.7	2.36	1	11.49	4.28	7.25	2.38
500-750	5.06	3.04	4.93	1.69	4.45	1.05	6.4	2.29	4	4.02	8.26	2.26	1.96	0.75	8.28	3.09	5.37	1.62
750-1000	4.09	2.58	4.8	2.25	3.32	1	5.28	2.67	1.87	3.13	5.49	1.94	1.91	0.9	6.64	3.32	4.39	1.23
1000-2500	15.56	6.07	17.92	5.14	14.6	5.28	19.25	5.77	2.73	4.93	20.12	5.83	7.88	3.68	15.64	6.73	13.78	6.61
2500-5000	14.45	6.18	16.92	5.06	12.42	3.97	17.68	6.6	0.58	2.24	11	6.77	8.34	2.29	6.08	6.71	11.91	5.79
5000-7500	7.15	4.15	12.06	7.23	9.44	5.78	11.43	7.1	0	0	7.21	8.25	5.41	3.75	0.57	2.21	4.86	4.36
7500-10000	4.43	4.66	5.69	5.56	6.49	4.1	3.07	3.34	0	0	4.09	4.73	5.96	5.29	1.58	4.24	7.35	2.87
10000-25000	18.71	11.85	14.17	9.76	16.88	7.88	11.7	9.86	0	0	0.94	2.5	16.06	6.54	0	0	12.34	9.08
25000-50000	8.43	11.84	4.26	8.47	11.22	8.32	2.1	3.25	0	0	0	0	11.18	12.43	0	0	13.75	13.08
50000-75000	3.01	8.32	0	0	0	0	0	0	0	0	0	0	34.75	18.01	0	0	4.92	13.81
75000-100000	0	0	0	0	0	0	0	0	0	0	0	0	0	0	0	0	0	0

Supplementary Table 2. Bacterial strains and plasmids used. Clinical strains were either collected from Ghent University Hospital (BS and OS) or selected from the international *P. aeruginosa* reference panel collection[1,2]. All strains were obtained from different patients.

Clinical strains		
P. aeruginosa isolates (accession number of genome sequence)	Description	Source or reference
PAO1 (NC_002516)	Wound isolate (Australia)	[3]
PA14 (NC_008463)	Burn wound isolate (US)	[4]
2192 (NZ_CH482384)	CF isolate (US)	[5]
AA2 (MCMJ00000000)	CF isolate (Germany)	[6,7]
AES-1R (MCML00000000)	CF isolate (Australia)	[7,8]
AMT0023-30 (MCMI00000000)	CF isolate (US)	[7,9]
AMT0060-3 (MCMZ00000000)	CF isolate (US)	[7,9]
AUS23 (MCMN00000000)	CF isolate (Australia)	[7,10]
BS1 (PRJNA1072279)	CF isolate (Belgium)	This study
BS2 (PRJNA1072279)	CF isolate (Belgium)	This study
BS3 (PRJNA1072279)	CF isolate (Belgium)	This study
BS5 (PRJNA1072279)	CF isolate (Belgium)	This study
BS6 (PRJNA1072279)	CF isolate (Belgium)	This study
BS9 (PRJNA1072279)	CF isolate (Belgium)	This study
BS11 (PRJNA1072279)	CF isolate (Belgium)	This study
BS14 (PRJNA1072279)	CF isolate (Belgium)	This study
C3719 (MCMM00000000)	CF isolate (UK)	[5,7]
CHA (MCMG00000000)	CF isolate (France)	[7,11]
DK2 (NC_018080)	CF isolate (Denmark)	[12]
IST27N (MCMW00000000)	CF isolate (Portugal)	[7,13]
KK1 (MCMB00000000)	CF isolate (Germany)	[6,7]
LESB58 (NC_011770)	CF isolate (UK)	[14]
LES400 (NZ_CP006982)	CF isolate (UK)	[15]
NH57388A (MCMT00000000)	CF isolate (Denmark)	[7,16]
NN2 (NZ_LT883143.1)	CF isolate (Germany)	[17,18]
OS1 (PRJNA1072279)	CF isolate (Belgium)	This study
OS2 (PRJNA1072279)	CF isolate (Belgium)	This study
OS3 (PRJNA1072279)	CF isolate (Belgium)	This study
OS4 (PRJNA1072279)	CF isolate (Belgium)	This study
RP1 (LNBU00000000)	CF isolate (Germany)	[19]
TBCF10389 (MCLZ00000000)	CF isolate (Germany)	[7,20]
U018A (MCMP00000000)	CF isolate (Australia)	[7,21]
P. aeruginosa PAO1 isogenic mutants		
ΔwspR	*ΔwspR* deletion mutant	This study
ΔsiaD	*ΔsiaD* deletion mutant	This study
Δpa5442	*Δpa5442* deletion mutant	This study
PAO1 WT/pHerd20T	PAO1 carrying empty pHerd20T, Cb^R	This study
ΔsiaD/pHerd20T	*ΔsiaD* mutant carrying empty pHerd20T, Cb^R	This study
PAO1 WT/pHerd20T-*siaD*	PAO1 carrying pHerd20T with *siaD* ORF inserted, Cb^R	This study
ΔsiaD /pHerd20T-*siaD*	*ΔsiaD* mutant carrying pHerd20T with *siaD* ORF inserted, Cb^R	This study
PAO1 *PsiaA::lacZ*	PAO1 with P*siaA-lacZ* and a Gm^R cassette inserted in the *att*::*Tn*7 site downstream of *glmS*	This study
GFP-labelled PA14		
PA14/pBK-miniTn7-gfp2	Chromosomal labelled PA14 with pBK-miniTn7-gfp2. Gm^R,	This study
E. coli strains		
HB101	K-12/B hybrid; Sm^r *recA thi pro leu hsdRM*$^+$	[22]
DH5α	F$^-$ ϕ80dlacZΔM15 Δ(lacZYA-argF)*U169 deoR recA1 endA1 hsdR17*(r_K^- m_K^+) *phoA supE44 λ*$^-$ *thi-1 gyrA96 relA1*	[23]
XL1Blue	*recA1 endA1 gyrA96 thi-1 hsdR17 supE44 relA1 lac* [*F' proAB lacI*^{q} *ZΔM15* Tn*10 (Tet*^{R}*)*]	[24]
Plasmids		
pBK-miniTn7-gfp2	pUC19-based delivery plasmid for miniTn7-*gfp2*. Gm^R, Cm^R, Ap^R, mob^+	[25]
pRK600	ColE1 RK2-Mob^+ RK2-Tra^+; helper plasmid; Cm^R	[24]
pUX-BF13	R6K replicon-based helper plasmid, providing the Tn7 transposition function in trans, $Ap^R mob^+$	[24]
pK18-Gm-mobsacB	Suicide knockout plasmid, Gm^R	[26]
pHerd20T	pUCP20T Plac replaced with 1.3-kb AflII-EcoRI fragment of araC-PBAD cassette, Amp^R	[27]

pHerd20T-*siaD*	pHerd20T plasmid carrying *siaD* ORF	This study
pUC18-mini-Tn7T-Gm-*lacZ*	Plasmid containing a mini-Tn7 system with single attTn7 site for integration in *P. aeruginosa* chromosome and a promoterless *lacZ*	[28]
pTNS2	Helper plasmid for integration of pUC18-mini-Tn7T-Gm	[28]
pUC18-mini-Tn7T-Gm-*PsiaA*-*lacZ*	pUC18-mini-Tn7T-Gm-lacZ containing the 500 bp upstream region of *siaA* fused to *lacZ*	This study

Supplementary Table 3. The presence of c-di-GMP related genes in different *P. aeruginosa* strains. +, gene is present; -, gene is absent.

	PAO1	PA14	2192	AA2	AES-1R	AMT0023-30	AMT0060-3	AUS23	C3719	CHA
siaD	+	+	+	+	+	+	+	+	+	+
pa0290	+	+	+	+	+	+	+	+	+	+
pa0338	+	+	+	+	+	+	+	+	+	+
pa0847	+	+	+	+	+	+	+	+	+	+
roeA	+	+	+	+	+	+	+	+	+	+
pa1851	+	+	+	+	+	+	+	+	+	+
pa2771	+	-	+	+	+	+	+	+	+	+
pa2870	+	+	+	+	+	+	+	+	+	+
pa3177	+	+	+	+	+	+	+	+	+	+
hsbD	+	+	+	+	+	+	+	+	+	+
sadC	+	+	+	+	+	+	+	+	+	+
pa4396	+	+	+	+	+	+	+	+	+	+
gcbA	+	+	+	+	+	+	+	+	+	+
pa4929	+	+	+	+	+	+	+	+	+	+
dgcH	+	+	+	+	+	+	+	+	+	+
tpbB	+	+	+	+	+	+	+	+	+	+
wspR	+	+	+	+	+	+	+	+	+	+
pa2133	+	+	+	+	+	+	+	+	+	+
pa2200	+	+	+	+	+	+	+	+	+	+
pa2572	+	+	+	+	+	+	+	+	+	+
arr	+	-	-	-	-	-	+	-	-	-
pa3825	+	+	+	+	+	+	+	+	+	+
rocR	+	+	+	+	+	+	+	+	+	+
pa4108	+	+	+	+	+	+	+	+	+	+
pa4781	+	+	+	+	+	+	+	+	+	+
pvrR	-	+	-	-	-	-	-	-	-	-

bifA	+	+	+	+	+	+	+	+	+	+
fimX	+	+	+	+	+	+	+	+	+	+
morA	+	+	+	+	+	+	+	+	+	+
mucR	+	+	+	+	+	+	+	+	+	+
pipA	+	+	+	+	+	+	+	+	+	+
rmcA	+	+	+	+	+	+	+	+	+	+
rbdA	+	+	+	+	+	+	+	+	+	+
yegE	+	+	+	+	+	+	+	+	+	+
pa1433	+	+	+	+	+	+	+	+	+	+
pa2072	+	+	+	+	+	+	+	+	+	+
pa2567	+	+	+	+	+	+	+	+	+	+
pa3258	+	+	+	+	+	+	+	+	+	+
nbdA	+	+	+	+	+	+	+	+	+	+
dipA	+	+	+	+	+	+	+	+	+	+
proE	+	+	+	+	+	+	+	+	+	+
pa5442	+	+	+	+	+	+	+	+	+	+

	**DK2**	**IST27**	**KK1**	**LES400**	**LESB58**	**NH57388A**	**NN2**	**RP1**	**TBCF10839**	**U018A**
siaD	+	+	+	+	+	+	+	+	+	+
pa0290	+	+	+	+	+	+	+	+	+	+
pa0338	+	+	+	+	+	+	+	+	+	+
pa0847	+	+	+	+	+	+	+	+	+	+
roeA	+	+	+	+	+	+	+	+	+	+
pa1851	+	+	+	+	+	+	+	+	+	+
pa2771	+	-	+	+	+	+	+	+	+	+
pa2870	+	+	+	+	+	+	+	+	+	+
pa3177	+	+	+	+	+	+	+	+	+	+
hsbD	+	+	+	+	+	+	+	+	+	+
sadC	+	+	+	+	+	+	+	+	+	+
pa4396	+	+	+	+	+	+	+	+	+	+
gcbA	+	+	+	+	+	+	+	+	+	+
pa4929	+	+	+	+	+	+	+	+	+	+

dgcH	+	+	+	+	+	+	+	+	+	+
tpbB	+	+	+	+	+	+	+	+	+	+
wspR	+	+	+	+	+	+	+	+	+	+
pa2133	+	+	+	+	+	+	+	+	+	+
pa2200	+	+	+	+	+	+	+	+	+	+
pa2572	+	+	+	+	+	+	+	+	+	+
arr	-	-	+	-	-	-	-	-	+	-
pa3825	+	+	+	+	+	+	+	+	+	+
rocR	+	+	+	+	+	+	+	+	+	+
pa4108	+	+	+	+	+	+	+	+	+	+
pa4781	+	+	+	+	+	+	+	+	+	+
pvrR	-	-	-	-	-	-	-	-	-	-
bifA	+	+	+	+	+	+	+	+	+	+
fimX	+	+	+	+	+	+	+	+	+	+
morA	+	+	+	+	+	+	+	+	+	+
mucR	+	+	+	+	+	+	+	+	+	+
pipA	+	+	+	+	+	+	+	+	+	+
rmcA	+	+	+	+	+	+	+	+	+	+
rbdA	+	+	+	+	+	+	+	+	+	+
yegE	+	+	+	+	+	+	+	+	+	+
pa1433	+	+	+	+	+	+	+	+	+	+
pa2072	+	+	+	+	+	+	+	+	+	+
pa2567	+	+	+	+	+	+	+	+	+	+
pa3258	+	+	+	+	+	+	+	+	+	+
nbdA	+	+	+	+	+	+	+	+	+	+
dipA	+	+	+	+	+	+	+	+	+	+
proE	+	+	+	+	+	+	+	+	+	+
pa5442	+	+	+	+	+	+	+	+	+	+

	**BS1**	**BS2**	**BS3**	**BS5**	**BS6**	**BS9**	**BS11**	**BS14**	**OS1**	**OS2**	**OS3**	**OS4**
siaD	+	+	+	+	+	+	+	+	+	+	+	+
pa0290	+	+	+	+	+	+	+	+	+	+	+	+
pa0338	+	+	+	+	+	+	+	+	+	+	+	+

pa0847	+	+	+	+	+	+	+	+	+	+	+	+
roeA	+	+	+	+	+	+	+	+	+	+	+	+
pa1851	+	+	+	+	+	+	+	+	+	+	+	+
pa2771	+	+	+	+	+	+	+	+	+	+	+	+
pa2870	+	+	+	+	+	+	+	+	+	+	+	+
pa3177	+	+	+	+	+	+	+	+	+	+	+	+
hsbD	+	+	+	+	+	+	+	+	+	+	+	+
sadC	+	+	+	+	+	+	+	+	+	+	+	+
pa4396	+	+	+	+	+	+	+	+	+	+	+	+
gcbA	+	+	+	+	+	+	+	+	+	+	+	+
pa4929	+	+	+	+	+	+	+	+	+	+	+	+
dgcH	+	+	+	+	+	+	+	+	+	+	+	+
tpbB	+	+	+	+	+	+	+	+	+	+	+	+
wspR	+	+	+	+	+	+	+	+	+	+	+	+
pa2133	+	+	+	+	+	+	+	+	+	+	+	+
pa2200	+	+	+	+	+	+	+	+	+	+	+	+
pa2572	+	+	+	+	+	+	+	+	+	+	+	+
arr	-	-	+	+	-	+	+	+	+	-	+	-
pa3825	+	+	+	+	+	+	+	+	+	+	+	+
rocR	+	+	+	+	+	+	+	+	+	+	+	+
pa4108	+	+	+	+	+	+	+	+	+	+	+	+
pa4781	+	+	+	+	+	+	+	+	+	+	+	+
pvrR	+	+	+	+	+	+	+	+	+	+	+	+
bifA	+	+	+	+	+	+	+	+	+	+	+	+
fimX	+	+	+	+	+	+	+	+	+	+	+	+
morA	+	+	+	+	+	+	+	+	+	+	+	+
mucR	+	+	+	+	+	+	+	+	+	+	+	+
pipA	+	+	+	+	+	+	+	+	+	+	+	+
rmcA	+	+	+	+	+	+	+	+	+	+	+	+
rbdA	+	+	+	+	+	+	+	+	+	+	+	+
yegE	+	+	+	+	+	+	+	+	+	+	+	+
pa1433	-	+	+	+	+	+	+	+	+	+	+	+

pa2072	+	+	+	+	+	+	+	+	+	+	+	+
pa2567	+	+	+	+	+	+	+	+	+	+	+	+
pa3258	+	+	+	+	+	+	+	+	+	+	+	+
nbdA	+	+	+	+	+	+	+	+	+	+	+	+
dipA	+	+	+	+	+	+	+	+	+	+	+	+
proE	+	+	+	+	+	+	+	+	+	+	+	+
pa5442	+	+	+	+	+	+	+	+	+	+	+	+

Supplementary Table 4. Strain cultivation time

Strain	Aggregate culture	Planktonic culture
PAO1	6 h	6 h
PA14	6 h	6 h
2192	6 h	6 h
AA2	6 h	6 h
AMT0023-30	6 h	6 h
AMT0060-3	6 h	6 h
CHA	6 h	6 h
IST27N	6 h	6 h
KK1	6 h	6 h
NN2	6 h	6 h
OS3	6 h	6 h
RP1	6 h	6 h
TBCF10839	6 h	6 h
U018A	6 h	6 h
AES-1R	9 h	9 h
AUS23	9 h	9 h
BS1	9 h	9 h
BS3	9 h	9 h
DK2	9 h	9 h
LESB58	9 h	9 h
NH57388A	9 h	9 h
OS2	9 h	9 h
BS2	12 h	12 h
BS5	12 h	12 h
BS9	12 h	12 h
OS1	12 h	12 h
C3719	18 h	9 h
LES400	18 h	18 h
OS4	18 h	18 h
BS6	24 h	24 h
BS11	24 h	9 h
BS14	24 h	24 h

Supplementary Table 5. MIC (µg/mL) of tobramycin and meropenem against different strains and mucoidy on PIA plates.

	**Tobramycin**	**Meropenem**	**Mucoidy on PIA**
PAO1	2	1	no
PA14	1	0.5	no
2192	1	0.5	no
AA2	2	1	no
AES-1R	8	8	no
AMT0023-30	1	0.5	no
AMT0060-3	1	0.25	no
AUS23	2	16	no
BS1	1	1	yes
BS2	1	0.125	no
BS3	16	4	yes
BS5	32	4	yes
BS6	2	1	yes
BS9	16	1	yes
BS11	2	4	yes
BS14	0.5	0.125	yes
CHA	2	0.5	no
C3719	4	64	no
DK2	32	32	no
IST27N	1	0.0625	no
KK1	1	0.5	no
LES400	8	8	no
LESB58	4	2	no
NH57388A	2	0.5	no
NN2	1	1	no
RP1	1	1	no
OS1	2	1	yes
OS2	2	0.25	yes
OS3	1	1	no
OS4	0.5	0.125	yes
TBCF10839	1	0.5	no
U018A	1	2	no

Supplementary Table 6. Primers used

Primers	Sequence 5'->3'
	Gene deletion
PAO1-wspR-up-F	AGCTCGGTACCCGGGATTGTTACCGTCGATATCGAGC
PAO1-wspR-up-R	CACCGGCTGTTCCATCAGTCGTATCAAATTGAATGCTGTTC
PAO1-wspR-dn-F	GAACAGCATTCAATTTGATACGACTGATGGAACAGCCGGTG
PAO1-wspR-dn-R	CGACGGCCAGTGCCAATCTCGATCTCGATGTTGTCGTC
PAO1-siaD-up-F	AGCTCGGTACCCGGGTCTCGGCCTGCTGGACATC
PAO1-siaD-up-R	CTGGACGCCTGAGGAGGCCAGTTGCTCCAGCGATTGCTG
PAO1-siaD-dn-F	CAGCAATCGCTGGAGCAACTGGCCTCCTCAGGCGTCCAG
PAO1-siaD-dn-R	CGACGGCCAGTGCCATTCGCCATGGAGATCATCAGC
PAO1-PA5442-up-F	AGCTCGGTACCCGGGATTGATCCCGAGCAACTGCTC
PAO1-PA5442-up-R	GAGTCGAAAGTGCTACAGCATACGAACACCAGCACCTGCTGGAG
PAO1-PA5442-dn-F	CTCCAGCAGGTGCTGGTGTTCGTATGCTGTAGCACTTTCGACTC
PAO1-PA5442-dn-R	CGACGGCCAGTGCCAAGCCTTGTCGACATTCTGC
	Sequencing
pk18-F	TGCTTCCGGCTCGTATGTTG
pk18-R	GCGAAAGGGGGATGTGCTG
pHerd20T-F	ATCGCAACTCTCTACTGTTTCT
pHerd20T-R	TGCAAGGCGATTAAGTTGGGT
PAO1-wspR-F	ATCGATGCAGCGGTGCAG
PAO1-wspR-R	AGCTGCGAAGTATACTGCACTTGC
PAO1-siaD-F	ACGACGAGTAGCCGACGATG
PAO1-siaD-R	TCGTCCTGACCCTGGAGTTG
PAO1-PA5442-F	ATCTGGAAGTTCTCGCTCAG
PAO1-PA5442-R	TTCGAGCTTCTCTTCCTCC
	Expression
SiaD-exp-F	ATGGGATCTGATAAGAATTCGTGCGGCTGGAGCGCATC
SiaD-exp-R	CGACGG CCA GTG CCA AGC TTT CAG CGC GCT GGA GCC GG
	lacZ reporter fusion
PsiaA_lacZ frag_F	GAATCCGTAATCATGGTCATGGCTATCCCTATCAGTTTCTC
PsiaA_lacZ frag_R	ATCATGCATGAGCTCACTAGGCGCAACCTGCTCGCCGG
PsiaA_lacZ vec_F	AGGCCGGCGAGCAGGTTGCGCCTAGTGAGCTCATGCATGATC
PsiaA_lacZ vec_R	GAAACTGATAGGGATAGCCATGACCATGATTACGGATTCAC
Tn7-GlmS	AATCTGGCCAAGTCGGTGAC
Tn7-R109	CAGCATAACTGGACTGATTTCAG
	RT-qPCR
qRT-siaD-F	CGCTACCAGCAGATGATG
qRT-siaD-R	GAGCGTTCGTTCTCCTC
qRT-pipA-F	ATCTCGCAATCGCCATCG
qRT-pipA-R	TTGCGGCGAAGAAGAACAG
qRT-pa0290-F	CAACGCGAATACCGCATC
qRT-pa0290-R	GCTTCTTGTCGGTGATGTC
qRT-pa0338-F	CATCCATCCCGAGGACTATC
qRT-pa0338-R	CGCTGATCCACAGGTAATCC
qRT-rmcA-F	AACCGCTTCACCTACGTC
qRT-rmcA-R	GTAGTGGATGGCGTACTCG
qRT-pa0847-F	CATGCTGTCGCCCATTAC
qRT-pa0847-R	TCCGACAAGAGCCAGATAC
qRT-rbdA-F	GGTGCTTCACGACATGAC
qRT-rbdA-R	TGCAGGCGATACTCGAAC
qRT-roeA-F	TGACCGGCCTGTTCAATC
qRT-roeA-R	GCGGTCGTTGATGTACTTG

qRT-yegE-F	GCCTGAACACCTGTTTCATC
qRT-yegE-R	GATCACCGAGACCAGGAAGG
qRT-pa1433-F	AGGCCTACCAGGACAGTCTC
qRT-pa1433-R	AGCCCGTTCAGGTCGTTCAG
qRT-pa1851-F	ATGATGGCCGAACAGGAC
qRT-pa1851-R	CGACATCCACCAGGATCAG
qRT-pa2072-F	CATCGAGCAGAGCCATTTC
qRT-pa2072-R	CCGGTTTCCCAGATAGACG
qRT-pa2133-F	GTATACCCGCGAAGCACGTC
qRT-pa2133-R	TCGTCCTCCTCGAGACCTTC
qRT-pa2200-F	TGTTGTTCGGCGTCATGCTG
qRT-pa2200-R	ATGGGCTGGTAGTGCACCTC
qRT-pa2567-F	CAATGGAACGCACGGAAC
qRT-pa2567-R	GGAAAGGTCGATGTCGTAGG
qRT-pa2572-F	CCTGCAACTGCTGGAAAG
qRT-pa2572-R	GTGAGCAGGATGCGAATG
qRT-pa2771-F	CCCTTCATCCGCTTCTACG
qRT-pa2771-R	ATTGAGCTGGCGTCCTTC
qRT-pa2870-F	CGTTCTACGCGTTGTTCTG
qRT-pa2870-R	TGGCGTTAAGCTCGATCAG
qRT-pa3177-F	GCCTGTTCTTCGAGGAAGTC
qRT-pa3177-R	TGTCATGGCTCAGGCGATAG
qRT-pa3258-F	ATTGCGACGAGAGCGTTC
qRT-pa3258-R	TTCGGAAAGCGAGAGGATG
qRT-nbdA-F	CAGATGGCTCGCTACGACAG
qRT-nbdA-R	TCGAGGTCGAGGAACATCAC
qRT-hsbD-F	ACGACTCCCGTTCCAATC
qRT-hsbD-R	GCTGTCCTCCAGCATGTAG
qRT-pa3825-F	ATCGAGTCGAGCGAAGTC
qRT-pa3825-R	TGGAACTTGCGCAGGTAG
qRT-pa4108-F	ACGTCTACGACGCGATCACC
qRT-pa4108-R	TTGACGAAGGCGCGGAACAC
qRT-sadC-F	CGGCATCTACCTGGTAGAG
qRT-sadC-R	TATGTAGCTGGCGAACAGG
qRT-pa4396-F	ACGGGCTCAACCTGAAG
qRT-pa4396-R	GCAGCAGTTCGTCGATG
qRT-pa4781-F	ATCTTCCGCGTATCGAGC
qRT-pa4781-R	GCCGATGTCATGCAACAG
qRT-gcbA-F	TCGATCAGCGAACTCAACC
qRT-gcbA-R	GTCCTTCAGTGCCAGGTAG
qRT-pa4929-F	CGACGAACTGCTGGAATAC
qRT-pa4929-R	GCACGTACCAGAAATAGGC
qRT-dipA-F	CAGTTGTCGCTGTCGAAG
qRT-dipA-R	GGCGAGTTTCTCGATGTG
qRT-proE-F	GACATGCAGTGGCTCAAC
qRT-proE-R	CGTGCTCTTCGATCAACC
qRT-pa5442-F	TCGCGGTGATCATGCTG
qRT-pa5442-R	AGGCGAAGGTGAGGAAG
qRT-dgcH-F	ATCGATGGCAGCCTGAAC
qRT-dgcH-R	CCGCAGGTATTCCTCGAAG
qRT-bifA-F	GGCGAGAACTTCGTCACCAC
qRT-bifA-R	GATCTTCGACAGCGGCTTGG
qRT-fimX-F	TCCTACATGGCGCTGTTC
qRT-fimX-R	TGGTAGCCCTTGAGGAAATC
qRT-morA-F	TCGTCCAGGTCAACGATTC
qRT-morA-R	TTGAGCTGGTTGGCTTCC
qRT-mucR-F	CAGCCGTACCAGATATCCC
qRT-mucR-R	TGGTCCTTGGCGTGATAC
qRT-RocR-F	CTGGTCGACAAGCTGTTC
qRT-RocR-R	ACCCAGTTGCGAAGGATG

qRT-tpbB-2-F	CTGATCGCCCGTTCCATCAG
qRT-tpbB-2-R	TAGACGATGGCGCTGGAGAC
qRT-wspR-F	GTGGCCAACCAGATCAAG
qRT-wspR-R	AGGACGATGATCGGGATG
qRT-proC-F	CAGGCCGGGCAGTTGCTGTC
qRT-proC-R	GGTCAGGCGCGAGGCTGTCT
qRT-ampC-F	AGATTCCCCTGCCTGTG
qRT-ampC-R	GCGGTGAAGGTCTTGCT
qRT-OprD-F	TCCGCAGGTAGCACTCA
qRT-OprD-R	AAGCCGGATTCATAGGTGG
qRT-recA-F	TCCGCAGGTAGCACTCAG
qRT-recA-R	AAGCCGGATTCATAGGTGG
qRT-gyrA-F	TGTGCTTTATGCCATGAGCGA
qRT-gyrA-R	TCCACCGAACCGAAGTTGC
qRT-fabD-F	GCATCCCTCGCATTCGTCT
qRT-fabD-R	GGCGCTCTTCAGGACCATT

References

1. Cullen, L. *et al.* Phenotypic characterization of an international *Pseudomonas aeruginosa* reference panel: strains of cystic fibrosis (CF) origin show less *in vivo* virulence than non-CF strains. *Microbiology (Reading)* **161**, 1961–1977 (2015).

2. De Soyza, A. *et al.* Developing an international *Pseudomonas aeruginosa* reference panel. *Microbiologyopen* **2**, 1010–1023 (2013).

3. Holloway, B. W. & Morgan, A. F. Genome organization in Pseudomonas. *Annu. Rev. Microbiol.* **40**, 79–105 (1986).

4. Rahme, L. G. *et al.* Use of model plant hosts to identify *Pseudomonas aeruginosa* virulence factors. *Proc. Natl. Acad. Sci. U. S. A.* **94**, 13245–13250 (1997).

5. Mathee, K. *et al.* Dynamics of *Pseudomonas aeruginosa* genome evolution. *Proc. Natl. Acad. Sci. U. S. A.* **105**, 3100–3105 (2008).

6. Lorè, N. I. *et al.* Cystic fibrosis-niche adaptation of *Pseudomonas aeruginosa* reduces virulence in multiple infection hosts. *PLoS One.* **7**, e35648 (2012).

7. Freschi, L. *et al.* Genomic characterisation of an international *Pseudomonas aeruginosa* reference panel indicates that the two major groups draw upon distinct mobile gene pools. *FEMS Microbiol. Lett.* **365** (2018).

8. Naughton, S. *et al. Pseudomonas aeruginosa* AES-1 exhibits increased virulence gene expression during chronic infection of cystic fibrosis lung. *PLoS One* **6**, e24526 (2011).

9. Mulcahy, L. R., Burns, J. L., Lory, S. & Lewis, K. Emergence of *Pseudomonas aeruginosa* strains producing high levels of persister cells in patients with cystic fibrosis. *J. Bacteriol.* **192**, 6191–6199 (2010).

10. O'Carroll, M. R. *et al.* Clonal strains of *Pseudomonas aeruginosa* in paediatric and adult cystic fibrosis units. *Eur. Respir. J.* **24**, 101–106 (2004).

11. Bezuidt, O. K. *et al.* Intraclonal genome diversity of *Pseudomonas aeruginosa* clones CHA and TB. *BMC Genomics* **14**, 416 (2013).

12. Yang, L. *et al.* Evolutionary dynamics of bacteria in a human host environment. *Proc. Natl. Acad. Sci. U. S. A.* **108**, 7481-7486 (2011).

13. Leitão, J. H., Alvim, T. & Sá-Correia, I. Ribotyping of *Pseudomonas aeruginosa* isolates from patients and water springs and genome fingerprinting of variants concerning mucoidy. *FEMS Immunol. Med. Microbiol.* **13**, 287–292 (1996).

14. Kukavica-Ibrulj, I. *et al. In vivo* growth of *Pseudomonas aeruginosa* strains PAO1 and PA14 and the hypervirulent strain LESB58 in a rat model of chronic lung infection. *J. Bacteriol.* **190**, 2804–2813 (2008).

15. Salunkhe, P. *et al.* A cystic fibrosis epidemic strain of *Pseudomonas aeruginosa* displays enhanced virulence and antimicrobial resistance. *J. Bacteriol.* **187**, 4908–4920 (2005).

16. Hoffmann, N. *et al.* Novel mouse model of chronic *Pseudomonas aeruginosa* lung infection mimicking cystic fibrosis. *Infect. Immun.* **73**, 2504–2514 (2005).

17. Cramer, N. *et al.* Microevolution of the major common *Pseudomonas aeruginosa* clones C and PA14 in cystic fibrosis lungs. *Environ. Microbiol.* **13**, 1690-1704 (2011).

18. Klockgether, J. *et al.* Structural genome variants of *Pseudomonas aeruginosa* clone C and PA14 strains. *Front. Microbiol.* **14**, 1095928 (2023).

19. Bianconi, I. *et al.* Comparative genomics and biological characterization of sequential *Pseudomonas aeruginosa* isolates from persistent airways infection. *BMC Genomics* **16**, 1105 (2015).

20. Klockgether, J. *et al.* Intraclonal diversity of the *Pseudomonas aeruginosa* cystic fibrosis airway isolates TBCF10839 and TBCF121838: distinct signatures of transcriptome, proteome, metabolome, adherence and pathogenicity despite an almost identical genome sequence. *Environ. Microbiol.* **15**, 191–210 (2013).

21. Pirnay, J.-P. *et al. Pseudomonas aeruginosa* population structure revisited. *PLoS One* **4**, e7740 (2009).

22. Kessler, B., de Lorenzo, V. & Timmis, K. N. A general system to integrate lacZ fusions into the chromosomes of gram-negative eubacteria: regulation of the Pm promoter of the TOL plasmid studied with all controlling elements in monocopy. *MGG Mol. Gen. Genet.* **233**, 293-301 (1992).

23. Chua, S. L. *et al.* Bis-(3'-5')-cyclic dimeric GMP regulates antimicrobial peptide resistance in *Pseudomonas aeruginosa*. *Antimicrob Agents Chemother* **57**, 2066–2075 (2013).

24. Anette, S. *et al.* Identification and Characterization of an N-Acylhomoserine Lactone-Dependent Quorum-Sensing System in *Pseudomonas putida* Strain IsoF. *Appl. Environ. Microbiol.* **68**, 6371–6382 (2002).

25. Koch, B., Jensen, L. E. & Nybroe, O. A panel of Tn7-based vectors for insertion of the gfp marker gene or for delivery of cloned DNA into Gram-negative bacteria at a neutral chromosomal site. *J. Microbiol. Methods.* **45**, 187-195 (2001).

26. Zhou, L., Wang, J. & Zhang, L. H. Modulation of bacterial type III secretion system by a spermidine transporter dependent signaling pathway. *PLoS One.* **2**, e1291 (2007).

27. Qiu, D., Damron, F. H., Mima, T., Schweizer, H. P. & Yu, H. D. PBAD-based shuttle vectors for functional analysis of toxic and highly regulated genes in Pseudomonas and Burkholderia spp. and other bacteria. *Appl. Environ. Microbiol.* **74**, 7422-7426 (2008).

28. Choi, K.-H. & Schweizer, H. P. mini-Tn7 insertion in bacteria with single attTn7 sites: example *Pseudomonas aeruginosa*. *Nat Protoc* **1**, 153–161 (2006).

nature portfolio

Corresponding author(s): Yuming Cai; Tom Coenye

Last updated by author(s): Yuming Cai; Tom Coenye

Editorial Policy Checklist

This form is used to ensure compliance with Nature Portfolio editorial policies related to research ethics and reproducibility. For further information, please see our editorial policies site. All relevant questions on the form must be answered.

Competing interests

Policy information about competing interests

In the interest of transparency and to help readers form their own judgements of potential bias, Nature Portfolio journals require authors to declare any competing financial and/or non-financial interest in relation to the work described in the submitted manuscript.

Competing interests declaration

[X] We declare that none of the authors have competing financial or non-financial interests as defined by Nature Portfolio.

[ ] We declare that one or more of the authors have a competing interest as defined by Nature Portfolio.

Authorship

Policy information about authorship

Prior to submission all listed authors must agree to all manuscript contents, the author list and its order and the author contribution statements. Any changes to the author list after submission must be approved by all authors.

[X] We have read the Nature Portfolio Authorship Policy and confirm that this manuscript complies.

Large Language Models (LLMs), such as ChatGPT, do not currently satisfy our authorship criteria. Notably an attribution of authorship carries with it accountability for the work, which cannot be effectively applied to LLMs. Use of an LLM should be properly documented in the Methods section (and if a Methods section is not available, in a suitable alternative part) of the manuscript.

[X] We confirm that the author list of this manuscript does not include any Large Language Models (LLMs).

Policy information about Authorship: inclusion & ethics in global research

All authors are encouraged to provide an "Inclusion & Ethics" statement where relevant.

[n/a] We have provided an "Inclusion & Ethics" statement.

Data availability

Policy information about availability of data

Data availability statement

All manuscripts must include a data availability statement. This statement should provide the following information, where applicable:

- Accession codes, unique identifiers, or web links for publicly available datasets
- A description of any restrictions on data availability
- For clinical datasets or third party data, please ensure that the statement adheres to our policy

[X] We have provided a full data availability statement in the manuscript.

Mandated accession codes (where applicable)

Confirm that all relevant data are deposited into a public repository and that accession codes are provided.

[X] All relevant accession codes are provided [ ] Accession codes will be available before publication [ ] No data with mandated deposition

Code availability

Policy information about availability of computer code

Code availability statement

For all studies using custom code or mathematical algorithm that is deemed central to the conclusions, the manuscript must include a statement under the heading "Code availability" describing how readers can access the code, including any access restrictions. Code availability statements should be provided as a separate section after the data availability statement but before the References.

n/a We have provided a full code availability statement in the manuscript

Data presentation

For all data presented in a plot, chart or other visual representation confirm that:

n/a	Confirmed	
[ ]	[X]	Individual data points are shown when possible, and always for $n \leq 10$
[ ]	[X]	The format shows data distribution clearly (e.g. dot plots, box-and-whisker plots)
[X]	[ ]	Box-plot elements are defined (e.g. center line, median; box limits, upper and lower quartiles; whiskers, 1.5x interquartile range; points, outliers)
[ ]	[X]	Clearly defined error bars are present and what they represent (SD, SE, CI) is noted

Image integrity

Policy information about image integrity

[X] We have read Nature Portfolio's image integrity policy and all images comply.

Unprocessed data must be provided upon request. Please double-check figure assembly to ensure that all panels are accurate (e.g. all labels are correct, no inadvertent duplications have occurred during preparation, etc.).

Where blots and gels are presented, please take particular care to ensure that lanes have not been spliced together, that loading controls are run on the same blot, and that unprocessed scans match the corresponding figures.

Additional policy considerations

Some types of research require additional policy disclosures. Please indicate whether each of these apply to your study. If you are not certain, please read the appropriate section before selecting a response.

Does not apply	Involved in the study	
[X]	[ ]	Macromolecular structural data
[X]	[ ]	Unique biological materials
[ ]	[X]	Research animals and/or animal-derived materials that require ethical approval
[X]	[ ]	Human embryos, gametes and/or stem cells
[X]	[ ]	Human research participants
[X]	[ ]	Clinical data
[X]	[ ]	Archaeological, geological, and palaeontological materials

Macromolecular structural data

Policy information about special considerations for specific types of data

Validation report

n/a We have provided an official validation report from wwPDB for all macromolecular structures studied.

Biological materials

Policy information about availability of materials

Obtaining biological materials: n/a

n/a We have described these restrictions in the manuscript. n/a We have described how to obtain all materials in the manuscript.

Research animals

Policy information about studies involving animals; ARRIVE guidelines recommended for reporting animal research

Ethical compliance

[X] We have complied with all relevant ethical regulations and include a statement affirming this in the manuscript.

Ethics committee

[X] We have disclosed the name(s) of the board and institution that approved the study protocol in the manuscript.

Human embryos, gametes and stem cells

Policy information about studies involving human embryos, gametes and stem cells

Manuscripts involving the use of human embryos, gametes or stem cells must include an ethics statement that provides the following information:
- The institutional and/or licensing committee(s) that approved the study protocol
- Confirmation that informed consent was obtained from all recipients and/or donors of cells or tissues
- The conditions for donating materials for the research

n/a We have read the Nature Portfolio policy on human embryos, gametes and stem cells and have complied with policy requirements.

Human research participants

Policy information about studies involving human research participants

Ethical compliance

n/a We have complied with all relevant ethical regulations and include a statement affirming this in the manuscript.

Ethics committee

Confirm that the manuscript states the name(s) of the board and/or institution that:

n/a Approved the study protocol -OR- n/a Provided guidelines for study procedures (if protocol approval is not required)

Informed consent

n/a We have obtained informed consent from all participants and this is noted in the manuscript.

Identifiable images

For publication of identifiable images of research participants, confirm that consent to publish was obtained and is noted in the Methods.

Authors must ensure that consent meets the conditions set out in the Nature Portfolio participant release form.

[ ] Yes [X] No identifiable images of human research participants

Clinical studies

Policy information about clinical studies

Clinical trial registration

n/a We have provided the trial registration number from ClinicalTrials.gov or an equivalent agency in the manuscript.

Phase 2 and 3 randomized controlled trials

We have provided the CONSORT checklist with your submission.

[ ] Yes [ ] No [X] Not a phase 2/3 randomized controlled trial

Tumor marker prognostic studies

We have followed the REMARK reporting guidelines.

[ ] Yes [ ] No [X] Not a tumor marker prognostic study

Archaeological, geological, and palaeontological materials

Policy information about studies involving archaeological, geological, and palaeontological materials

n/a ☐ We affirm that archaeological, geological, and palaeontological materials samples were collected (and, where applicable, exported) in a responsible manner and in accordance with relevant permits and local laws, and that this information is detailed within the manuscript.

I certify that all the above information is complete and correct.

Typed signature Yuming Cai Date 07/08/2024

This checklist template is licensed under a Creative Commons Attribution 4.0 International License, which permits use, sharing, adaptation, distribution and reproduction in any medium or format, as long as you give appropriate credit to the original author(s) and the source, provide a link to the Creative Commons license, and indicate if changes were made. The images or other third party material in this article are included in the article's Creative Commons license, unless indicated otherwise in a credit line to the material. If material is not included in the article's Creative Commons license and your intended use is not permitted by statutory regulation or exceeds the permitted use, you will need to obtain permission directly from the copyright holder. To view a copy of this license, visit http://creativecommons.org/licenses/by/4.0/
